# Characteristics of Selected Antioxidative and Bioactive Compounds in Meat and Animal Origin Products

**DOI:** 10.3390/antiox8090335

**Published:** 2019-08-22

**Authors:** Bartosz Kulczyński, Andrzej Sidor, Anna Gramza-Michałowska

**Affiliations:** Department of Gastronomy Sciences and Functional Foods, Faculty of Food Science and Nutrition, Poznań University of Life Sciences, Wojska Polskiego 31, 60-624 Poznań, Poland

**Keywords:** antioxidants, taurine, l-carnitine, choline, alpha-lipoic acid, conjugated linoleic acid, glutathione, creatine, coenzyme Q10, peptides, meat, health-promoting properties

## Abstract

Meat and meat products have a high nutritional value. Besides major components, meat is rich in bioactive components, primarily taurine, l-carnitine, choline, alpha-lipoic acid, conjugated linoleic acid, glutathione, creatine, coenzyme Q10 and bioactive peptides. Many studies have reported their antioxidant and health-promoting properties connected with their lipid-lowering, antihypertensive, anti-inflammatory, immunomodulatory activity and protecting the organism against oxidative stress. The antioxidant activity of meat components results, among others, from the capability of scavenging reactive oxygen and nitrogen species, forming complexes with metal ions and protecting cells against damage. This review is focused to gather accurate information about meat components with antioxidant and biological activity.

## 1. Introduction

Increasing interest in the relationship between the diet, nutrients and health has contributed to the development of new directions in research focused on the determination of the effect of specific compounds on physiological functions in living organisms. Being aware of the importance of proper nutrition in disease prevention and control consumers search for food products characterized by high nutritive value. The foundation for the adequate supply of essential nutrients is provided by a balanced, varied diet, based on diverse groups of foods, including cereals, vegetables, fruit, fish, meat and oils. However, the health effect of consumed meat, particularly red meat, is increasingly often considered disputable. The perception of meat and its products as raw materials having a negative effect on health results, among other things, from its relatively high contents of cholesterol, saturated fatty acids and sodium [1]. Recently the body of evidence indicating that high consumption of red meat and its processed products may be correlated with the development of certain chronic diseases, e.g., obesity, cardiovascular disease and cancer. At the same time, it is absolutely clear that meat products are important sources of antioxidants and numerous essential nutrients, either not found in other groups of food products or found in limited amounts. These nutrients include e.g., high-quality protein, microelements (iron, zinc, selenium, manganese) and vitamins (A, B12, folic acid) [2,3]. Moreover, various types of meat are rich in bioactive compounds [4], which may be defined as nutritive and non-nutritive substances, natural or synthetic nutrients, which may potentially enhance, inhibit or modify physiological and metabolic functions of the human organism [5]. Antioxidants are natural or synthetic substances possessing the ability to inhibit or delay oxidation process at relatively low concentrations. They have been divided into primary and secondary antioxidants. First ones are preventive antioxidants and donators, which activity depends on peroxides inactivation by annexation to free radicals of fatty acid. Secondary antioxidants, called donators, protect substrate by, e.g., scavenging of singlet oxygen, absorbing the UV radiation, synergistic activity, decomposition of peroxides and nonradical products. Antioxidants positively affect the shelf life, nutritional and sensory properties of a product [6]. Over the last decades, we have been observing an increase in the global production of animal origin products, which has been connected with the growing demand for meat and its processed products. As reported in literature, in the years 1961–2009 the greatest increase in production (11-fold) was recorded for poultry meat from 8891 thousand tons up to 93,818 thousand tons. An increase in production was also observed for pork (from 24,666 to 106,269 thousand tons), beef (from 28,594 to 66,065 thousand tons), and lamb and mutton as well as goat meat (from 5854 to 13,106 thousand tons). It is estimated that the global production of poultry meat in 2020 in comparison to 2009 will increase by 31%, for pork, lamb and mutton as well as goat meat it will be by approximately 20%, and by 12% for beef. In the years 1961/63–2007/09 total consumption of meat worldwide increased by 75%, which is equivalent to the mean consumption of 41.5 kg meat per capita annually. The greatest increase was observed in Asia (by 416%) and Europe (by 56%) [7]. According to the data of FAO (Food and Agriculture Organization of the United Nations), mean global beef consumption in 2012 was 6.52 kg per capita, for lamb and mutton it was 1.7 kg per capita, pork—12.48 kg per capita, while for poultry it was 13.15 kg per capita, respectively. Current data indicates that in 2024, meat consumption is expected to decrease by almost 2% in developed countries in comparison to 2012. In turn, in developing countries meat consumption will increase by approximately 6% [8].

**Figure 1:**
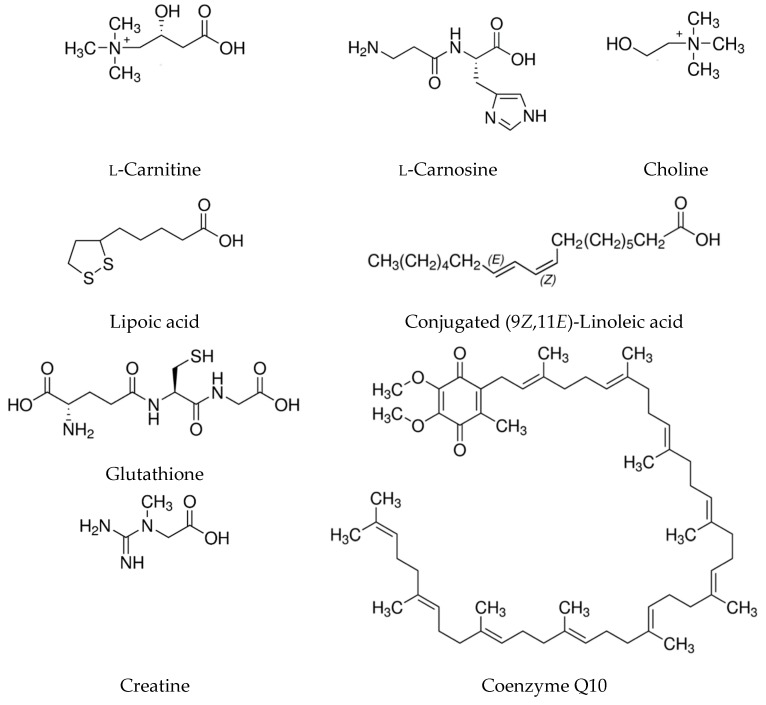
Chemical structures of selected bioactive compounds found in meat and its products.

The following paper provides a synthetic and systematic review of the current literature on the subject of selected antioxidative and bioactive compounds found in meat. Figure 1 presents the chemical structures of the compounds found in meat and its products characterized in the following paper. Detailed data on the effects of meat components intake by animals and humans is presented in Table 1 and Table 2.

### 1.1. l-carnitine

l-carnitine (3-hydroxy-4-*N*,*N*,*N*-trimethylaminobutyrate) is a water-soluble quaternary amine, which is synthetized in vivo from lysine and methionine in kidneys and the liver of mammals [46]. l-carnitine is described as a conditionally essential nutrient for humans and animals. Approximately 75% carnitine intake is supplied with the diet, while 25% intake is synthesized in the body [46]. It is estimated that the daily intake of this nutrient is around 20–200 mg [80], while the human organism synthesizes approximately 20 mg l-carnitine a day [81]. Apart from the above-mentioned amino acids, this compound may be produced only in the presence of vitamin C, iron, vitamin B6 and niacin, which catalyzes many reactions [82]. Bioavailability of l-carnitine changes depending on the composition of consumed food. In vegetarians, who are adapted to foodstuffs poor in carnitine, its availability is higher (66–86%) than in the individuals whose diet contains meat (54–72%) [83]. It needs to be stressed that bioavailability of l-carnitine coming from dietary supplements is as low as 15–18% [80]. Carnitine is found in two stereoisomers forms, l and d, while biological activity is observed only for the l-isomer [84]. d-carnitine is biologically inactive and it is not found in nature [85]. l-carnitine was isolated for the first time by Russian researchers from muscle tissue (*Latin carnus*), thus producing its name. Due to the similarity of its action to that of the B vitamins, it was labelled vitamin BT [80]. This name was based on the results of experiments, in which a deficit of this nutrient in the diet was shown to cause the accumulation of fat in larvae of *Tenebrio molitor* [80]. The human pool of l-carnitine is approximately 20 g, of which 98% are located in the cardiac and skeletal muscles, while 1.4% in the liver and kidneys, and 0.6% in the extracellular fluid [82]. The most important dietary sources of l-carnitine include animal origin products: beef, pork, chicken breast, fish, lamb, as well as products such as milk, eggs and cheeses (Table 3) [85]. This compound may be found in slight amounts also in plant origin products: nuts, seeds, legumes, cereal products or vegetables [85]. l-carnitine performs several significant functions in living organisms. It plays an important role in the generation of energy by mediating in the fatty acids oxidation reactions in the mitochondrial matrix. It participates in the activated fatty acids (acylo-CoA) transport from the cytoplasm across the mitochondrial membrane, thus stimulating processes of β-oxidation, at the same time increasing thermogenesis [85]. l-carnitine is also involved in the metabolism of branched-chain amino acids: valine, leucine and isoleucine. By binding with their metabolic products (α-ketoacids), it transports them to the liver, where they are oxidized, or they are substrates in the process of gluconeogenesis [80]. There are numerous studies, which indicate the antioxidant effect of l-carnitine. In a 40-day experiment, in which rats fed a high-fat diet were administered l-carnitine dissolved in water (75 mg/L), an increase was observed in the antioxidant enzymes activity found in the plasma of experimental animals, i.e., glutathione, peroxide dismutase, glutathione peroxidase and catalase [45]. Similar results were reported by Sepand et al., who showed that oral intake of 300 mg/kg acetyl-l-carnitine for a period of 28 days caused an increase in the activity of antioxidant enzymes (catalase, peroxide dismutase and glutathione transferase) in the plasma and liver, kidneys, brain, heart, lungs tissues of rats, which at the same time were administered arsenic compounds. At the same time the activity of aspartate transferase, alanine transaminase and lactate dehydrogenase was observed to decrease in blood serum [44]. Additionally, studies conducted by Lee et al. showed that a 12-week supplementation of l-carnitine at 1000 mg/day caused a statistically significant reduction of malondialdehyde levels in patients with coronary heart disease [12]. Antioxidant properties of l-carnitine were also confirmed by in vitro studies. This compound was shown to be capable of scavenging hydrogen peroxides as well as chelating metal ions (cadmium and iron) [80]. Some studies also indicated the hypolipidemic effects of l-carnitine. Elgazzar et al. [46] showed that intake of l -carnitine in various doses of 25, 50 and 100 mg/kg caused a decrease in serum total cholesterol, triglycerides, LDL (low-density lipoproteins) and HDL (high-density lipoproteins) cholesterol levels in experimental rats. At the same time an increase was recorded in HDL cholesterol concentration. The observed effect was dependent on the level of that compound. An experiment conducted on rats with streptozotocin-induced type II diabetes showed that daily administration of l-carnitine at 125 or 250 mg/kg (equivalent to approximately 100–200 g beef) reduced both plasma and hepatic triacylglycerol levels [48]. The hypolipidemic effect was also recorded in a study conducted by Mohammed-Jawad et al. [13], who administered 1000 mg l-carnitine for 8 weeks to patients with type II diabetes. The authors of that experiment recorded reduced serum concentrations of total cholesterol (by 9.14%), LDL cholesterol (by 8.3%) and lipoprotein (by 37.51%). No statistically significant changes were observed in the LDL cholesterol level. It was also observed that l-carnitine exhibits anti-inflammatory action. A study by Lee et al. [11] showed that supplementation with that compound reduced inflammatory markers levels: interleukin 6 (IL-6), C-reactive protein (CRP) and tumor necrosis factor (TNF-α). Moreover, it was reported that intake of this compound may prevent fatty liver [84]. A low plasma carnitine level in comparison to the guidelines indicates a deficiency of that compound. An insufficient l-carnitine level may be related with its decreased intake with the diet, reduced in vivo synthesis caused e.g., by hepatic dysfunctions and excessive losses of that nutrient during accelerated diuresis, hemodialysis or diarrhea [85]. Symptoms and consequences of carnitine deficiency include particularly myopathies, cardiomyopathy, heart and liver failure. An insufficient l-carnitine intake also causes increased glycolysis, resulting in the development of hypoglycemia and hypoketonemia [80].

### 1.2. l-carnosine

l-carnosine (beta-alanyl-l-histidine) is a water-soluble endogenous dipeptide composed of β-alanine and l-histidine [87]. Its biosynthesis involves carnosine synthetase and molecules of adenosine triphosphate (ATP) [88]. This compound is naturally found in the brain, kidneys and skeletal muscles of fish, birds and mammals [89]. It is commonly accepted that carnosine content in the organism is sex-dependent (with a higher level in males), age (with its concentration decreasing with age) and the diet (with the vegetarian diet connected with a lower carnosine concentration in skeleton muscles). It is also suggested that the synthesis of carnosine in muscles is dependent on the availability of β-alanine in the body [90]. Literature data indicate that a high histidine intake is correlated with increased carnosine levels in tissues [91]. In animals, such stress factors as trauma, shock, hunger or injection have a negative effect on the level of carnosine in muscle tissue [90]. It is estimated that carnosine may account for 0.2–0.5% mass of certain muscles [90]. Its contents in animal origin products are given in Table 4 [92]. l-carnosine exhibits antioxidant properties, it is capable of scavenging reactive oxygen and nitrogen species [90]. It also forms complexes with metal ions (cobalt, iron, zinc and copper) protecting cells against damage [89,90,91]. Research results suggest that l-carnosine in combination with α-tocopherol exhibits a synergistic action [88]. In an experiment conducted on rats it was shown that supplementing feed with 0.5% l-carnosine caused an increase in the plasma, skin and liver peroxide dismutase activity in comparison to control animals. At the same time the activity of glutathione peroxidase in liver tissue was observed to increase and the concentration of malondialdehyde was found to decrease in plasma and skin [93]. Both in vitro and in vivo studies confirmed that l-carnosine inhibits lipid peroxidation [91]. In turn, an experiment conducted on rats with ethanol-induced liver damage showed that the administration of l-carnosine contributed to an increase the antioxidant enzyme activity (glutathione) and a decrease liver malondialdehyde concentration [94]. Carnosine is considered to act as an inhibitor of the angiotensin converting enzyme (ACE) [88]. Results of other studies performed on animals suggest a neuroprotective action of carnosine [95]. It is assumed that this compound may play a role in treatment of Alzheimer’s disease due to its capacity to quench toxicity of beta-amyloid [89]. Carnosine may also affect lipid metabolism. Kim et al. showed that supplementation of feed with carnosine caused a decrease serum LDL cholesterol concentration and an increase in HDL cholesterol concentration. At the same time no statistically significant differences were recorded in the concentrations of total cholesterol and triacylglycerols between animals administered carnosine and animals not receiving that additive [93]. In turn, experiments on obese rats showed that daily administration of 30 mg/kg carnosine (equivalent to approximately 6.5 g pork ham) for 24 weeks caused a statistically significant reduction of total cholesterol and triacylglycerols concentrations [96].

l-Carnosine has the ability to counteract the adverse consequences of increased oxidative stress and oxidative transformation as a result of aging, metabolic diseases and age-related illnesses. Carnosine injections given to aging rats aged 20 months and rats with d galactose-induced aging slowed oxidative modification of protein (PC), lipids (TBARS, conjugate-DC), the formation of advanced glycation products (AGEs) and advanced protein oxidation products (AOPP), while reducing production of reactive oxygen species (ROS) [49,52]. A similar effect occurred in rats with diabetes induced by high fat diet (HFD) and streptozotocin (STZ). Carnosine reduced the formation of ROS, TBARS, PC, AOPP and AGE. Despite the decrease in ROS concentration, glutathione (GSH), superoxide dismutase (SOD), catalase (CAT) and glutathione peroxidase (GPx) activity remained unchanged. No changes in mRNA, SOD and GPx expression in the liver suggests that carnosine does not stimulate the production of exogenous antioxidants. Carnosine did not affect the glucose and HbA1c levels in rats [50,51]. In contrast to the above studies on diabetic rats, patients taking only hyperglycaemic drugs found a decrease in blood glucose and HbA1c concentration after receiving 1 g of carnosine daily for 12 weeks. Furthermore, prooxidative and proinflammatory processes have been inhibited [17,18]. Other study showed that the administration of 1 g of carnosine daily for three months to diabetic patients led to improvement of the lipid profile, total antioxidant capacity of the body and lowering HbA1c level [16].

### 1.3. Choline

Choline (2-Hydroxy-*N*,*N*,*N*-trimethylethan-1-aminium) is an essential nutrient [97], which sources include first of all animal origin products such as eggs, beef and pork (Table 5) [98,99]. Mean daily intake of choline was 8.4 mg/kg (for men) and 6.7 mg/kg (for women) [98]. Literature data give the recommended choline intake at 550 mg/d for males and 425 mg/d for females [100]. This compound may also be synthesized de novo in the body and it is found primarily (95%) in the form of phosphatidylcholine. However, it needs to be stressed here that endogenous synthesis of choline is insufficient to meet the requirement for this nutrient in humans [99,101]. Phosphatidylethanolamine is a substrate in the biosynthesis of choline. This reaction is catalyzed by phosphatidylethanolamine N-methyltransferase. In the liver, choline is metabolized to betaine [99]. Choline and its derivatives are structural elements of lipoproteins and membrane lipids. They are also precursors of a neurotransmitter—acetylcholine (Ach) [99]. Moreover, choline is an important source of monocarbon units, particularly during folic acid deficiency. Choline supplementation in humans reduces the total homocysteine concentration (tHcy) [99]. It is suggested that a high choline intake and its high level in blood plasma in pregnant women reduces the risk of the neural tube defects in newborns. According to the research results presented by Shaw et al. [102], women found within the lowest quartile of choline intake had a fourfold greater risk of giving birth to children with neural tube defects in comparison to women within the highest intake quartile. Research results indicate that individuals whose diet was rich in choline and betaine had the lowest concentrations of inflammatory markers: C-reactive protein (CRP), interleukin 6 (IL-6) and tumor necrosis factor (TNF-α) [103]. Choline deficiency contributes to the development of various disorders in animals and humans. This may lead to the development of fatty liver, hepatocyte death as well as skeletal muscle damage [101]. A low level of choline may also result in a reduction of mitochondrial membrane potential and a decreased production of adenosine triphosphate (ATP) [101]. Research indicated that rats fed a choline-deficient diet suffered from lipid beta-oxidation disorders [101]. Interestingly, population studies involving 5918 men and women indicated that a concentration of free choline in the body was correlated with a high anxiety level [104].

### 1.4. Alpha-Lipoic Acid

Alpha-lipoic acid ((R)-5-(1,2-Dithiolan-3-yl)pentanoic acid or 6,8-Dithiooctanoic acid) is a natural compound found both in plants and animals, which is synthesized in the mitochondria from octanoic acid and cysteine [105]. Lipoic acid is found in the form of two enantiomers: R-enantiomer and S-enantiomer, while the naturally found lipoic acid is the R-form [106]. Alpha-lipoic acid was discovered in 1937, while it was isolated for the first time in 1951 [107]. Dietary sources of this compound include vegetables (spinach, broccoli, tomatoes), offal (mainly the heart, kidneys and liver) and dietary supplements [107,108]. Contents of Alpha-lipoic acid in various meats are presented in Table 5. Alpha-lipoic acid and its reduced form are ascribed strong antioxidant properties. It is capable of scavenging ROS (hydrogen peroxide, hydroxyl radicals, singlet oxygen, peroxynitrite, hypochlorous acid), regeneration of endogenous antioxidants such as e.g., vitamin E, vitamin C, glutathione from their oxidized forms as well as chelation of metal ions (e.g., copper, manganese, lead, zinc, lead and iron) [107,108,109]. Results reported by numerous authors confirm the effect of alpha-lipoic acid on carbohydrate and lipid metabolism in various organisms. An experiment in which rats kept on a high-fat diet were administered intraperitoneally 54 mg/kg alpha-lipoic acid for a period of eight weeks, showed that the addition of this compound reduced blood glucose concentration and resulted in a decrease of the Homeostatic Model Assessment–Insulin Resistance index (HOMA-IR). In that study, reduced serum, total cholesterol, triacylglycerols and very low density lipoprotein (VLDL) cholesterol levels and increased HDL cholesterol concentration were also recorded in the group of animals receiving alpha-lipoic acid [110]. Moreover, a decrease was also observed in malonate L-dialdehyde levels in liver tissues [110]. In a similar study conducted by Murali et al. it was reported that supplementing high-fat diet of broiler chickens with alpha-lipoic acid at 100 mg/kg feed contributed to a statistically significant reduction of LDL cholesterol concentration and an increase in HDL cholesterol level. However, no differences were found in the total cholesterol, triacylglycerols and VLDL cholesterol concentrations [111]. An improvement was found in such parameters as glucose level, insulin concentration and HOMA-IR values in an experiment conducted by Yang et al., with a more advantageous effect recorded at the application of 100 mg/kg/d alpha-lipoic acid that at the intake of 200 mg/kg/d [112]. A reduction of blood glucose and insulin concentration was observed in an experiment involving diabetic rats administered 200 mg/kg alpha-lipoic acid daily. In that study an increased concentration of adiponectin and decreased levels of total cholesterol, free fatty acids and triacylglycerols were also recorded [113]. In turn, an experiment performed by Morakinyo et al. showed that intake of alpha-lipoic acid caused an increase of plasma antioxidant enzymes activity, peroxide dismutase and glutathione, in rats with streptozotocin-induced diabetes. An increase was also recorded in the catalase (CAT) activity; however, that result was not significant statistically [114]. An increased SOD and GSH activity, as well as increased levels of vitamins C and E in the liver were observed in rats with type II diabetes following a 12-week intraperitoneal administration of alpha-lipoic acid at 35 mg/kg [109]. Some studies also indicate the hypotensive effect of alpha-lipoic acid. Properties of that compound reducing arterial blood pressure may be correlated with an improved insulin sensitivity of cells [115], inhibited overproduction of endothelin-1 (a vasostructural substance) and increased synthesis of nitric oxide (a vasodilatation factor) [106]. Available literature sources also indicate the immunomodulatory effect of alpha-lipoic acid. In experiments conducted on mice with multiple sclerosis it was found that alpha-lipoic acid inhibits demyelination, being the underlying cause of the disease. It is generally accepted that this effect results e.g., from the reduced secretion of proinflammatory cytokines: interleukin 4 (IL-4) and interferon-γ (INF-γ) by T-lymphocytes, a reduced influx of T-lymphocytes and macrophages to the central nervous system [116].

Alpha-lipoic acid in type 2 diabetic rats prevented hepatic steatosis by reducing the oxidation reaction, increasing the activity of antioxidant enzymes and expressing the Nrf2 transcription factor (nuclear erythroid 2-related factor) that plays key roles in protecting cells from the damaging effects of oxidative stress. Other factors protecting against the harmful effects of the disease were reduction of inflammatory processes and lowering values for typical diabetes markers, such as: glucose, cholesterol, non-HDL and triglyceride (TG) levels [61].

### 1.5. Conjugated Linoleic Acid (CLA)

Conjugated dienes of linoleic acid, also called conjugated linoleic acid (CLA) refer jointly to position groups (c8, c10; c9, c11; c10, c12 and c11, c13) and geometric groups defining the configuration on the double bond in the molecule (*cis/cis; cis/trans; trans/cis* and *trans/trans*) of isomers of octadecadienoic acid (linoleic acid), which contain conjugated double bonds in their carbon chain [118,119]. Sources of CLA include, first of all, meat of ruminants and dairy products. These acids are synthesized in the rumen of e.g., cattle, sheep, goats and deer as a results of biotransformation of unsaturated fatty acids such as oleic and linoleic acids, while they are accumulated primarily in their adipose tissue [120]. These acids are produced involving bacteria e.g., *Butyrinvibrio fibrisolvens*, found in the animal stomachs [117]. Recently it was shown that other bacteria are also capable of synthesizing CLA isomers. These microorganisms include first of all bacteria from the genera *Bifidobacterium*, *Lactobacillus*, *Lactococcus* and *Propionibacterium* [121]. The presence of CLA in meat is dependent on the diet of the animals, while its amount is relatively low, amounting to 2–5 mg/g total fat content (Table 5) [117]. It is estimated that in the Western population intake of this nutrient amounts to 50–500 mg/day [122]. On the commercial scale CLA is synthesized from oils rich in linoleic acid (e.g., quality sunflower oil) via alkaline isomerization [123]. Conjugated dienes of linoleic acid exhibit high biological activity. There are numerous studies indicating that CLA intake has an advantageous effect on body mass reduction. Both in vivo and in vitro experiments suggest that this effect may be correlated with enhanced lipolysis in adipocytes and increased beta-oxidation of fatty acids in adipocytes and skeletal muscle cells [117]. Daily CLA supplementation at 6.4 mg/d for 36 weeks contributed to a reduction of the Body Mass Index (BMI) and total fat mass [124]. Long-term (annual) CLA supplementation with formulations containing free fatty acids or triacylglycerols lasting one year led to a decrease in body weight, adipose tissue and increase in the share of lean body mass. The changes also included a decrease in LDL and HDL concentration [22]. Bulut et al. observed a decrease in TG, LDL and VLDL in slightly overweight people performing exercises, which was connected with an increase in lipoprotein lipase activity [24]. Some of the research does not confirm the biological activity of CLA [25,27]. There was also no conclusive evidence of the effect of CLA on platelet function in patients with mild to moderate cardiovascular disease. Significant changes were only in the expression of the P-selectin cell adhesion molecule and the sex-dependent increase in platelet fibrinogen binding in men and inhibitory effects on collagen-induced aggregation in women [23]. According to Gaullier et al. CLA increased the risk factors for cardiovascular disease: an increase in the concentration of leukocytes, thrombocytes and lipoprotein a, which is a strongly atherogenic factor [22].

CLA may also influence carbohydrate metabolism. Experiments conducted on obese diabetic rats supplied evidence confirming the advantageous effect of CLA on improvement of glucose tolerance. At the same time it was observed that the obtained effect was comparable to the effect of troglitazone (an oral antidiabetic drug) [125]. Some studies indicate hypotensive properties of CLA. Administration of synthetic CLA was found to prevent the development of arterial hypertension induced by obesity in experimental rats. A similar effect was also observed in normal weight rats with spontaneous hypertension [126]. The hypotensive effect was confirmed in clinical trials. It was shown that the supplementation of 4.5 g CLA for eight weeks caused a decrease in systolic and diastolic blood pressure [122]. Conjugated dienes of linoleic acid are also ascribed anti-inflammatory and immunomodulatory activity. It was confirmed that CLA both in vivo and in vitro quenches the secretion of proinflammatory cytokines, e.g., the tumor necrosis factor (TNF-α) and interleukin 8 (IL-8), and enhances the secretion of the transforming growth factor (TGF-β1), i.e., an anti-inflammatory cytokine. CLA was also found to increase the immunoglobulins A (IgA), G (IgG) and M (IgM) production, at the simultaneous synthesis inhibition of immunoglobulin E (IgE) [127]. As it was shown, CLA has an advantageous effect on the serum lipid profile. Studies involving diabetic patients showed that the daily intake of a mixture of two CLA isomers (c9t11 and t10c12) at 3 g caused an increase in the HDL cholesterol concentration, while at the same time it decreased the LDL: HDL ratio [127]. In another study, a decrease in the serum total cholesterol and LDL cholesterol concentration was recorded in obese patients after a 24-month CLA supplementation [128]. At the same time no differences were observed in the triglycerides, triacylglycerols or HDL cholesterol levels. It also needs to be stressed the fact that some research results suggest a lack of hypolipidemic effect of CLA. Valeille et al. showed that the intake of milk fat rich in ruminal acid (*cis*-9,*trans*-11-octadecadienoic acid) may inhibit atherogenesis [129]. The authors reported a reduction of cholesterol levels in arterial tissues of experimental hamsters and improved blood cholesterol transport. What is more, they also observed that CLA protects LDL molecules against oxidation.

### 1.6. Glutathione

Reduced glutathione (γ-glutamyl-cysteinyl-glycine; GSH) is a low molecular weight, water soluble tripeptide composed of amino acids: cysteine, glycine and glutamic acid [33]. This compound is found both in plant and animal cells. It is synthesized primarily in hepatocytes. Next glutathione is transported with blood to other tissues. This highest concentrations are detected mainly in kidneys, the brain, erythrocytes, leukocytes, lungs, the heart, intestines and muscles. It needs to be stressed that a high protein intake causes a considerable increase in the concentration of glutathione, while hyperthyroidism considerably reduced its concentration (by 40%). Literature sources report that its level decreases also with age as a result of the decreasing g-glutamylcysteine synthetase activity. The diet has a marked effect on the content of this nutrient. The most abundant sources of glutathione include vegetables, fruit and cooked meat. Other products such as eggs, oils, beverages, dairy and cereal products are poor in that compound (Table 6) [130]. The amount of glutathione is also dependent on the presence of methionine and cysteine, contained in large amounts in meat (beef, poultry) and animal origin products such as eggs and milk (cow, ewe and goat) [130]. The most important function of glutathione in living organisms is to protect cells against oxidative stress. This compound efficiently scavenges reactive oxygen species, e.g., hydroxyl radicals, hydrogen peroxides, lipid peroxides and superoxide anions. Glutathione also participates in detoxification of lipid oxidation products, primarily malondialdehyde and 4-hydroxy-2-nonenal [130]. A reduced glutathione level is observed in the development of many diseases, such as diabetes, alcoholism, AIDS and neurodegenerative diseases (Alzheimer’s, Parkinson’s). In Parkinson’s patients the glutathione concentration in the grey matter is by 40% lower than in healthy individuals. At the same time, it needs to be stressed that a lower concentration of glutathione is desirable when temporary immunosuppression is required during transplantation procedures. At the same time, a low concentration of this compound in cancer cells increases their sensitivity to radio- and chemotherapy [130]. GSH supplementation in healthy people influenced the increase in its whole blood concentration, erythrocytes, plasma, peripheral blood mononuclear cells (PBMC) and buccal cells. GSH stimulated the body’s immune function by potentiating the cytotoxicity of natural killer cells and lymphocyte proliferation. At the same time, it limited the oxidation reactions [29,30]. On the other hand, in Allen and Bradley studies, GSH (1 g for four weeks) did not change the level of oxidative stress [33]. Three hundred milligrams of GSH per day during four months influenced the desired changes of selected NAFLD (nonalcoholic fatty liver disease) markers, including the level of ferritin, non-esterified fatty acids and ALT (alanine aminotransferase) [32]. GSH has the potential to counteract the development of cardiovascular diseases. In patients with CVD risk factors, GSH helped in reducing the TC, LDL, but for unclear reasons also HDL [31]. Liposomal GSH reducing the oxidative stress and pressure attenuates reperfusion injury in rabbit hearts [63]. In the case of apolipoprotein E-deficient mice (atherosclerotic mice), liposomal GSH reduced the area of atherosclerotic lesions and modulated cholesterol metabolism in macrophages [62].

### 1.7. Taurine

Taurine (2-aminoethanesulfonic acid) is a sulfur amino acid, which is found in the free form in the body, as it does not form peptide bonds. In mammals it is contained in many tissues: the brain, retina, liver, skeletal muscles, cardiac muscle, blood platelets and leukocytes [131]. The compound was isolated for the first time in 1827 from bull’s bile [86]. Its name was derived from the Latin name of that animal species (*Bos taurus*) [132]. For humans the primary sources of taurine include the diet rich in meat and animal origin products (Table 3). The highest amounts of taurine were detected in shellfish, fish and turkey meat [132]. Plant origin products contain trace amounts of this nutrient, as a result low serum taurine concentration is recorded in vegetarians [132]. The capacity for endogenous synthesis of taurine varies between species. In adult humans, the mean daily synthesis of this compound amounts to 50–125 mg. It is estimated that its content in the body of 70 kg is approximately 70 g. In turn, biosynthesis of this compound in cats is very low and covers the body requirement in only several percent [132,133]. Observations showed that a taurine-deficient diet leads to retinopathy in those animals [132]. Methionine and cysteine are taurine precursors. Vitamin B6 is required for its synthesis to occur. This process takes place mainly in the brain and liver. However, it is assumed that the endogenous production of taurine is insufficient to cover the systemic requirement [134]. Taurine is excreted both with urine and feces [119]. It needs to be stressed that a protein-rich diet promotes its elimination from the body due to the increased cholecystokinin secretion [132]. As a consequence of the significant role played by taurine, its deficiency is connected with many pathological conditions. A long-term low intake of this nutrient may be correlated with various disorders: retinopathy, growth and development retardation, cardiovascular disorders, dysfunction of the central nervous system or hepatic dysfunction [86]. Available literature indicates a multitude of functions served by taurine in living organisms. This compound is a component of bile, in which it is conjugated with bile acids. Conjugation of these acids with taurine results in its increased polarity and solubility [132]. Taurine is ascribed antioxidant properties. It is capable of binding with strong oxidants, mainly hypochlorous acid, forming taurine mono- and dichloroamine, i.e., compounds, which are less reactive than the above-mentioned acid [132]. Studies conducted by Murakami et al. [72] provided evidence suggesting the effect of taurine on lipid metabolism. The authors of that experiment, conducted on hamsters with hypercholesterolemia induced by a high-fat diet, observed that animals supplemented with taurine had lower plasma total cholesterol, triglycerides and phospholipids concentrations. At the same taurine intake reduced hepatic levels of free and esterified cholesterol. Taurine was also found to play a considerable role in carbohydrate metabolism. Carneiro et al. [135] showed that taurine controls homeostasis of glucose via two mechanisms: (1) by regulating the expression of genes involved in glucose-stimulated secretion of insulin, and (2) by increasing insulin sensitivity of peripheral tissues. An experiment conducted on rats showed that taurine enhances glycogen synthesis as well as intensifies glycolysis and glucose uptake in the liver and heart of experimental animals [136]. Moreover, it was reported that this amino acid may have a potential inhibitory effect on the absorption of glucose from the alimentary tract, as it inhibits the activity of sodium glucose transporter (SGLT-1) [132]. Some studies also confirmed the hypotensive effect of taurine. An experiment, in which rats with arterial hypertension induced by a high-fructose diet were administered taurine, showed that an addition of the amino acid inhibits the increase in arterial blood pressure [71]. It is believed that the hypotensive effect of taurine results from its inhibitory effect on the hyperactive sympathetic system [137]. Additionally, taurine exhibits natriuretic and diuretic properties [138]. Literature data emphasizes the protective role of taurine towards the retina. It is suggested that this compound protects the retina against oxidative stress caused by visible radiation. Moreover, research results indicate that taurine participates in differentiation of retinal cells [132]. Taurine is also ascribed the function of a neurotransmitter and neuromodulator. A high content of this amino acid in the cerebral cortex, hippocampus, cerebellum and the hypothalamus seems to confirm these assumptions [139].

### 1.8. Bioactive Peptides

Bioactive peptides are short sequences containing approximately 2–20 amino acids, which have an advantageous physiological effect upon intake [140]. Their molecular mass is typically below 6 kDa [141]. They are derived from food, both plant and animal origin. However, most bioactive peptides are obtained from animal origin products such as milk, eggs, bovine blood, collagen, gelatin or various fish species (e.g., salmon, tuna, herring) and other marine organisms [142,143]. An example of a bioactive peptide was described for the first time in 1950, when Mellander suggested that phosphorylated peptides derived from casein improve bone calcification in rachitic infants [144]. In recent years we have been observing a considerable interest in meat as a source of quality peptides [144]. Bioactive peptides are obtained from proteins as precursors using various methods, including proteolysis taking place in the intestinal tract, chemical or enzymatic hydrolysis run in vitro and during food processing, or as a result of microbial fermentation [144]. The process yielding specific peptides involves various types of proteases originating from plant tissues (e.g., physin, papain, bromelain), animal tissues (e.g., pepsin, trypsin, chymotrypsin), as well as microbial cells (proteinase K, collagenase, subtilisin) [145]. Moreover, in recent years new methods have been developed based on molecular genetic engineering, which facilitate the synthesis of peptides when their amino acid sequence is known [144]. It needs to be stressed that the activity of bioactive peptides depends on the composition of amino acids, their specific sequence, the type of amino acid at the *N*- and *C*-terminal ends, hydrophobic and hydrophilic properties as well as the mass and length of the peptide chain [144]. In available literature the largest number of studies have concerned the effect of bioactive peptides on the arterial blood pressure reduction. These peptides exhibit the angiotensin-converting-enzyme inhibitors (ACEI) activity [146]. Arihara et al., in myosine isolated from pork, detected the presence of two pentapeptides with MNPPK (Met-Asn-Pro-Pro-Lys) and ITTNP (Ile-Thr-Thr-Asn-Pro) sequences inhibiting the angiotensin convertase action. The authors of that experiment observed that the intake of these peptides at 1 mg/kg body mass in rats caused a decrease in systolic blood pressure by 23.4 and 21.0 mmHg [147]. In turn, Jang and Lee identified hexapeptide (VLAQYK) originating from sarcoplasmic proteins of beef, which also exhibited an action resembling that of angiotensin-converting-enzyme inhibitors [148]. Peptides inhibiting the angiotensin convertase activity were also isolated as a result of chicken collagen hydrolysis using protease produced by a fungus *Aspergillus oryzae* [149]. What is of interest, biopeptides also exhibit antioxidant activity [150]. Bernardini et al. [151] showed that peptides obtained from beef brisket using enzymatic hydrolysis are capable of scavenging synthetic DPPH (2,2-diphenyl-1-picrylhydrazyl) radicals, as well as reducing and chelating iron ions. In turn, Saiga et al. showed that hydrolysates of pork myofibrillar proteins produced using proteases (papain and actinase E) exhibited high antioxidant activity in the system simulating iron ion induced oxidation of linoleic acid [152]. In turn, in another experiment Jang et al. [153] reported antibacterial properties of four peptides originating from hydrolysates of beef sarcoplasmic proteins. Isolated biopeptides, depending on their type and concentration, had an inhibitory effect on growth of pathogenic microorganisms such as *Escherichia coli*, *Pseudomonas aeruginosa*, *Salmonella typhimurium*, *Staphylococcus aureus*, *Bacillus cereus* and *Listeria monocytogenes*. Mora et al. researches indicate that the aging of beef meat for four weeks under chilled storage resulted positively on the formation of bioactive peptides, which could have a significant impact on the promotion of the maturing meat consumption [154].

### 1.9. Coenzyme Q10

Coenzyme Q10 (2-[(2E,6E,10E,14E,18E,22E,26E,30E,34E)-3,7,11,15,19,23,27,31,35,39-Decamethyltetraconta-2,6,10,14,18,22,26,30,34,38-decaenyl]-5,6-dimethoxy-3-methylcyclohexa-2,5-diene-1,4-dione [155], also referred to as ubiquinone, is a fat-soluble vitamin-like compound. It was isolated from mitochondria of the bovine heart muscle in 1957. It is found throughout the body, with its greatest concentration recorded in such organs as muscles, the spleen, pancreas, heart, liver, kidneys and the brain [156]. The best dietary source of coenzyme Q10 include meat products, fish and vegetable oils (Table 7). This substance is also found in smaller amounts in vegetables (parsley, spinach, napa cabbage), fruit (avocado, grapes) or nuts (peanuts, walnuts, hazelnuts, pistachios) [81]. An enhanced coenzyme production is observed in the presence of vitamins B2, B6, B12, folic and pantothenic acids [157]. Coenzyme Q10 may also be synthesized artificially and in such a form it is used as a dietary supplement. It needs to be stressed that coenzyme Q10 ranks third among bestselling dietary supplements in the USA, immediately after omega-3 acids and multivitamin preparations, and its daily intake allowance is estimated at 3–6 mg [81]. The primary role of coenzyme Q10 is to mediate in the mitochondrial electron transport system [158]. Ubiquinone is essential for the high-energy ATP molecules production. Numerous medical conditions are associated with coenzyme Q10 deficiency, e.g., osteoporosis, fibromyalgia, cardiovascular diseases (atherosclerosis, arterial hypertension, cardiomyopathies), neurodegenerative diseases (Alzheimer’s, Parkinson’s, multiple sclerosis), diabetes, periodontitis, nephropathies or male infertility [157,159,160]. Deficiency of that nutrient may result from e.g., its disturbed synthesis caused by malnutrition (such as an insufficient intake of vitamin B6, which is an essential cofactor in biosynthesis reactions), genetic or acquired defects affecting the coenzyme synthesis, increased requirements for this nutrient caused by underlying diseases 160]. It seems that high doses of coenzyme Q10 (1200 mg/day for adults and 10 mg/kg/day for children) are safe and well-tolerated [160]. Ubiquinone is found in all cellular membranes and in the body it also serves the function of an antioxidant, which amount and efficiency exceed those of other compounds exhibiting antioxidant properties [159]. It protects DNA against oxidative damage caused by the action of free radicals, while it also exhibits the capacity of regenerating other antioxidants, such as tocopherol or ascorbic acid, and prevents lipid peroxidation found in the inner mitochondrial membrane [159]. It is also suggested that ubiquinol (the reduced form of ubiquinone) participates in scavenging free radicals formed as a result of metabolism of certain xenobiotics (e.g., anthracycline antibiotics) [157]. Randomized placebo controlled trials showed that supplementation with coenzyme Q10 at 300 mg/day (corresponding to approximately 2.4 kg pork hearts) for 12 weeks caused an increase in the serum antioxidant enzymes activity of coronary artery disease (CAD) patients [21]. At the same time a statistically significant reduction was recorded in the levels of proinflammatory factors: interleukin 6 (IL-6) and the tumor necrosis factor (TNF-α). Meta-analysis conducted by Rosenfeldt et al. [161] indicated hypotensive properties of coenzyme Q10. It was shown that intake of coenzyme Q10 by arterial hypertension patients caused a systolic and diastolic blood pressure reduction (by 17 mmHg and 10 mmHg, respectively). Results of many studies also supplied a considerable body of evidence confirming the hypolipidemic effect of ubiquinone. El Haleem et al. [53] observed that the administration of coenzyme Q10 to rats kept on a high-fat diet contributed to a decrease in plasma total cholesterol, triglycerides and LDL cholesterol concentrations at a simultaneous significant increase in the HDL cholesterol level. In addition, it reduced the intensity of oxidative stress by limiting lipid peroxidation and increasing the concentration of antioxidant enzymes. On the other hand, in trials involving hyperlipidemic patients it was observed that coenzyme Q10 supplementation led to an increase in HDL cholesterol concentration, whereas no statistically significant changes were recorded in plasma levels of total cholesterol, LDL cholesterol and triglycerides [162].

The dose of 120 mg of coenzyme Q10 administered daily for 24 weeks allowed patients with dyslipidemia to lower BMI, blood pressure, triglycerides and LDL cholesterol, as well as glucose and insulin levels [20]. Coenzyme Q10 may have potential application in diabetic patients by regulating the metabolism of lipids and carbohydrates [13] and increasing total antioxidant potential along with inhibiting the development of inflammation of diabetic hemodialysis patients [19]. Coenzyme Q10 showed activity in patients with coronary artery disease treated with statins. Supplementation with coenzyme Q10 influenced the increase of its amount in plasma, increase of vitamin E concentrations, antioxidant enzymes and lowered C-reactive protein (CRP) inflammation marker [21].

### 1.10. Creatine

Creatine (Methylguanidoacetic acid) is a nitrogen compound naturally found in animal tissues [163]. Its name is derived from the Greek word “kreas” meaning meat [44]. Creatine is synthesized mainly in the liver, kidneys and the pancreas from amino acids: glycine, methionine and arginine. Production of this compounds requires also the presence of enzymes: l-arginine: glycine amidinotransferase (AGAT), guanidinoacetate *N*-methyltransferase (GAMT) and methionine adenosyltransferase (MAT). Skeletal muscles contain approximately 95% creatine pool in the body. The other 5% are located mainly in the brain, liver, kidneys and testes [164]. The pool in an average young man of 70 kg body mass is estimated at 120–140 g [165]. Approximately 60% creatine is found in the phosphorylated form as phosphocreatine [PCr] [166]. Endogenous synthesis supplied approximately 1 g creatine daily [165]. This compound may also be supplied with the diet. Individuals consuming red meat and seafood may provide approximately 1–2 g creatine daily with their diet [167]. Contents of creatine in selected meat products and fish are given in Table 7. Dietary supplements are sources of creatine for athletes [164]. It seems that the most important physiological role served by creatine is connected with its participation in the production of energy. This compound is responsible for the maintenance of an adequate intracellular level of adenosine triphosphate (ATP) in skeletal muscles during their intensive contractions [164]. Moreover, it may potentially accelerate the increase in muscle mass and force by increasing the activity of satellite cells, production of anabolic hormones, expression of myogenic transcription factors and by reducing protein catabolism and oxidative stress [167].

### 1.11. Meat Consumption and Health Controversy

According to International Agency for Research on Cancer (IARC), the cancer agency of the World Health Organization report, regular consumption of red meat can cause cancer [168]. The heterocyclic aromatic amines N-nitroso-compounds and polycyclic aromatic hydrocarbons generated during meat processing in high temperatures are the main substances responsible of red meat potential carcinogenicity. However, according to Domingo and Nadal the potential role of a number of environmental chemical contaminants, which are already present in raw or unprocessed meat, is not often taken into a consideration [169]. It has been found, that although some cooking techniques affect the levels of chemical contaminants in food, their content depends mainly on the initial amount in the raw food product. A relationship between red meat, or processed meat consumption and cancer incidence was observed for colorectal, pancreatic and prostate cancers, but the evidence in this regard is not sufficiently clear. Results of latest research show that a number of gaps still exist, and it is essential to establish the mechanisms leading to the increased risk of cancers, type 2 diabetes or cardiovascular diseases arising from red and processed meat consumption [170,171,172]. The increasing popularity of vegetarian diet and the results of studies indicating the relationship between the consumption of raw or processed red meat and cancer occurrence may affect its participation in the supply of some nutrients. For these health concerns, lowering the consumption of red meat and processed meat in general, seems to be significant trend in high-consuming countries. As a result, however, we can expect new challenges related to inadequate intake of vitamin B12, protein intake below requirements for the children and elderly, as well as low Zn intake in relation to child growth [173,174].

The crucial thing about food research is that diets and foods have usually complex composition and its beneficial or adverse health effects could not be attributed to one or a limited number of specific nutrients or compounds. Moreover, equally important is the interactive effect of meat with other food components on the gut microbiome, also in terms of dietary patterns should be investigated. Therefore, it is important to know and understand the mechanisms determining meat composition and the impact of its consumption on health and disease. However, it should be noted that moderate consumption of meat and meat products as part of a balanced diet is not a threat to health and life of the consumer.

## 2. Conclusions

An increasing number of studies published recently indicates that meat and meat products are rich not only in essential nutrients, such as quality protein, heme iron, zinc or vitamin B12, but also physiologically active compounds influencing consumers’ health. Bioactive compounds found in meat include first of all l-carnitine, l-carnosine, choline, lipoic acid, conjugated dienes of linoleic acid (CLA) glutathione, taurine, coenzyme Q10, creatine as well as biopeptides. Available literature indicates the multifaceted functions served by these compounds in animals and humans. They participate in carbohydrate and lipid metabolism. They are also compounds mediating in mitochondrial electron transport and they are responsible for energy generation. They also exhibit a broad spectrum of health-promoting effects, as they e.g., improve lipid parameters, reduce arterial blood pressure, reduce inflammatory conditions by inhibiting the production of proinflammatory cytokines, participate in detoxification, show antibacterial properties, lead to the reduction of fat mass, as well as protect the organism against oxidative stress. Some of the compounds discussed above are components of dietary supplements used in adjunctive therapy of certain medical conditions or to improve physical fitness. Contents of bioactive compounds in meat products depend first of all on the animal species and body part from which a given product was obtained. It needs to be stressed that some of the studies were based on the administration of specific compounds to animals or humans in amounts impossible to provide with typical diets. Nevertheless, meat products are not only their primary, but also natural sources in the human diet. The consumer is looking for healthy meat products, with high nutritional value that are easy to prepare and produced without negative impact on the environment. Despite recent adverse meat ratings, it appears to be one of the few food products that can be designed in vitro in the laboratory. Owing to modern science, this is becoming more and more real, however its nutritional and culinary value is not entirely predictable yet. Additionally, the quality of meat depends not only on the breeding method but also of gastronomic treatment applied. Until now, meat is an ingredient of many functional products and its healthy properties are appreciated by the consumers.

## Figures and Tables

**Table 1:** Effects of meat components intake–in vivo studies performed on humans.

Component	Experimental Model	Study Group	Treatment	Effects	Reference
**Acetyl-l-carnitine**	Randomized, phase III, double-blind, placebo-controlled trial	Patients with hypertension, T2DM and dyslipidemia on background statin therapy (*n* = 229) were randomized to the placebo (*n* = 113) and acetyl-l-carnitine (*n* = 116) groups.	2000 mg acetyl-l-carnitine/d (2 × 1000 mg capsules) for 6 months	BW↔, BMI↔, SBP↓, DBP↔, mean BP↓, glucose↔, HbA1c↑, insulin↔, HOMA-IR↓, GDR↔, TC↑, HDL↓, LDL↔, TG↔, Lp(a)↔, serum creatinine ↔, albuminuria↔, GFR↔	Parvanova et al. [9]
**l-carnitine** (Eva Pharma, Egypt)	Parallel randomized controlled prospective	T2DM patients on glimepiride (*n* = 72) were randomized to glimepiride group (*n* = 34), and glimepiride + l-carnitine group (*n* = 38).	2 g l-carnitine/d (1 g twice daily) for 6 months	BMI↔, SBP↔, DBP↔, fasting glucose↓, postprandial blood glucose↓, HbA1c↓, insulin↓, HOMA-IR↓, IRAPe↑, TNF-α↓, Visfatin↓, TC↓, TG↓, HDL↑, LDL↓	El-sheikh, El-Haggar and Elbedewy [10]
**l-carnitine** commercially available capsules (New Health Taiwan Co., Ltd.).	Single blind, randomized, parallel, placebo-controlled trial	Patients with coronary artery disease (*n* = 47) were randomly assigned to the placebo (*n* = 24) and l-carnitine (*n* = 23) groups.	1000 mg l-carnitine/d (2 × 500 mg capsules) for 12 weeks	• CRP↓, IL-6↓, TNF-α↓; • levels of inflammation markers were negatively correlated with the levels of LC and antioxidant enzymes activities (SOD, GPx);	Lee et al. [11]
**L-carnitine** Commercially available capsules (New Health Taiwan Co., Ltd.).	Single blind, randomized, parallel, placebo-controlled trial	Patients with coronary artery disease (*n* = 47) were randomly assigned to the placebo (*n* = 24) and L-carnitine (*n* = 23) groups.	1000 mg l-carnitine/d (2 × 500 mg capsules) for 12 weeks	• CAT↑, GPx↑, SOD↑, MDA↓, l-carnitine↑ • level of L-carnitine was significantly correlated with CAT and SOD activities	Lee et al. [12]
**l-carnitine** tablets (Ultimate Nutrition Company, USA) **Coenzyme Q10** soft gel (Vitane’s Nature Company, USA)	Randomized controlled single center clinical trial	Type 2 diabetes patients (*n* = 75) who treated with oral antidiabetic drugs metformin and sulfonylurea) were randomly assigned into l-carnitine, coenzyme Q10 and control groups.	l-carnitine 1000 mg tablet once daily for 8 weeks	glucose↓, HbA1c↔, TC↓, LDL↓, HDL↔, Lp(a)↓	Mohammed-Jawad et al. [13]
			150 mg coenzyme Q10 soft gel daily (2 × 75 mg) for 8 weeks	glucose↓, HbA1c↓, TC↓, LDL↓, HDL↔, Lp(a)↓	
**l-carnitine** (Lanling Pharmaceutical CO., LTD, China)	Randomized, single-blinded, placebo-controlled clinical study	Patients with MetS (*n* = 30) were randomly allocated into l-carnitine (*n* = 15) and control (*n* = 15) groups	4 g l-carnitine infusion daily (2 g twice a day) for 7 days	• BW↓, BMI↓, WC↓, HC↓, WHR↓, SBP↔, DBP↔, TC↔,TG↓, HDL↓, LDL↑, ApoA1↓, ApoB↑, ApoA1/ ApoB↓, Lp(a)↑, glucose↓, insulin↓, HOMA-IR↓, CRP↔, UA↑, FFA↑, AST↑, ALT↔, GGT↓ • hunger score in the L-carnitine group was decreased. • reduction physical and mental fatigue and fatigue severity scores during starvatio: improved physical fatigue (l-carnitine vs. control, *p* < 0.001), mental fatigue (l-carnitine vs. control, *p* = 0.001), and fatigue severity (l-carnitine vs. control, *p* < 0.001).	Zhang et al. [14]
**Carnosine** (Flamma S.p.A, Italy)	Pilot randomized, double-blind, placebo-controlled trial	Overweight and obese, non-diabetic individuals (*n* = 30), were assigned to carnosine and placebo groups	2 g/day (2 × 1 g) for 12 weeks	adipsin↔, leptin↔, resistin↓	Baye et al. [15]
**l-Carnosine** capsules (NOW FOODS Company for Natural Products manufactured by GMP Pharma, USA)	Randomized, double-blinded, placebo-controlled trial	Patients (*n* = 90) with type 1 diabetes, aged 9 to 18 years with at least 5 years disease duration, active diabetic nephropathy in the form of microalbuminuria were randomly assigned into carnosine (*n* = 45), or matching placeb group (*n* = 45). Patients in both groups received oral captopril 25 mg tablet	1 g/d (2 × 500 mg capsule) administered orally for 3 months	BW↔, BMI↔, SBP↔, DBP↔, glucose↔, TG↓, TC↓, HDL↑, HbA1c↓, creatinine↔, UACR↓, Alpha 1-microglobulin↓, TAC↑, MDA↓, serum carnosine↑	Elbarbary et al. [16]
**l-Carnosine** capsules (Myprotein, UK and Ireland)	Double-blind, randomized, parallel-design, clinical trial	Oral agents for controlling hyperglycemia (*n* = 54) were randomly assigned into carnosine (*n* = 27) and placebo (*n* = 27) group	1 g/d (2 × 500 mg capsules) after a meal for 12 weeks	BW↔, BMI↔, WC↔, BFM↓, FFM↑, SBP↓, DBP↔, glucose↓, HbA1c↓, insulin↓, HOMA-IR↔, HOMA-β↔, TG↓, TC↔, LDL↔, HDL↔, CML↓, pentosidine↓, s-RAGE↔, TNF-α↓, IL-6↓, IL-1β↔	Houjeghani, Kheirouri, Faraji and Jafarabadi [17]
**l-Carnosine** capsules (Myprotein, UK and Ireland)	Double-blind, randomized, parallel designed, clinical trial	Patients with T2DM, using only oral agents for controlling hyperglycemia (*n* = 54) were randomly assigned into carnosine (*n* = 27) and placebo (*n* = 27) group	1 g/d (2 × 500 mg capsules) after a meal for 12 weeks	glucose↓, CAT↑, SOD↔, MDA↓, PC↓	Houjeghani, Kheirouri, Faraji et al. [18]
**Coenzyme Q10** (Zahravi Company, Iran)	Randomized, double-blinded, placebo-controlled clinical trial	Diabetic hemodialysis patients were randomly assigned into coenzyme Q10 (*n* = 30) or placebo (*n* = 30) groups	120 mg coenzyme Q10/d (60 mg twice a day) for 12 weeks	TAC↑, GSH↔, MDA↔, CRP↓, NO↑	Fallah, Askari, Soleimani et al. [19]
**Coenzyme Q10** soft gel (BY-Health Co Ltd., China)	Randomized, double-blinded, placebo-controlled trial	Dyslipidemic subjects without taking any hypoglycemic or hypolipidemic drugs (*n* = 101) were randomly assigned to the placebo (*n* = 50) or coenzyme Q10 (*n* = 51) groups.	120 mg coenzyme Q10 daily (2 softgels 30 mg coenzyme Q10 each twice a day) for 24 weeks	BW↔, HC↓, WC↔, BMI↓, SBP↓, DBP↓, TC↔, TG↓, LDL↓, HDL↔, non HDL↔, ApoA1↑, ApoB↔, ApoA1/ApoB↑, glucose↓, insulin↓, HOMA-IR↓, CRP↔, TAC↑, AST↔, ALT↔, GGT↔, urea↔, creatinine↔, UA↔	Zhang, Yang, Guoet al. [20]
**Coenzyme Q10** commercially available capsules (New Health Taiwan Co., Ltd., Taiwan)	Single blinded, randomized, parallel, placebo-controlled study	Patients with coronary artery disease with statins therapy for at least 1 month (*n* = 51) were randomly assigned to the placebo (*n* = 24) or coenzyme Q10 (*n* = 27) groups.	300 mg coenzyme Q10/d for 12 weeks	coenzyme Q10↑, vitamin E↑, SOD↑, CAT↑, GPx↑, CRP↔, TNF-α↓, IL-6↔, adiponectin↔	Lee, Tseng, Yen and Lin [21]
**Conjugated linoleic acid** - CLA free fatty acids (FFA): *cis*-9, *trans*-11 isomer (39 g/100 g) and the *trans*-10, *cis*-12 isomer (41 g/100 g) - CLA triacylglycerols: *cis*-9, *trans*-11 isomer (38 g/100 g) and the *trans*-10, *cis*-12 isomer (38 g/100 g) (Natural Lipids, Norway)	Randomized, double-blind, placebo-controlled study	Healthy overweight volunteer men and women with BMI 25–30 kg/m^2^ (n = 180) were randomly assigned to placebo (*n* = 59), CLA-FFA (*n* = 61) or CLA-triacylglycerol (*n* = 60)	4.5 g 80% CLA-FFA (3.6 g active CLA isomers) or 4.5 g 76% CLA triacylglycerols (3.4 g active isomers) for 12 months	BW↓, BMI↓, BFM↓, LBM↑, BMM↓, diet daily intake↓, HbA1c↑, glucose↔, TG↔, TC↔, HDL↓, LDL↓, Lp(a)↑, leukocytes↑, thrombocytes↑, ALT↔, AST↑	Gaullier, Halse, Høye et al. [22]
**Conjugated linoleic acid** CLA80:20 capsules (Stepan Specialty Products BV, Netherlands) each containing 1 g of oil and 0.05% v/v Tocoblend TM L50 IP (IOI Loders Croklaan, NL) as anti-oxidant	Double-blind, randomized, cross-over, baseline, and placebo controlled human intervention study	Healthy subjects at low and moderate cardiovascular risk (*n* = 45) assigned to placebo (*n* = 23) or CLA (*n* = 22) groups	Four capsules daily for two weeks, crossing over to the other treatment arm after a wash-out of at least four weeks. The dose (4 g/day) provided 2.5 g/day 9c,11t-CLA or 1.1% of energy	• plasma FA: 16:0↔, 18:0↔, 18:1 t11↔, 18:1 c9↔, 18:2 n6↔, 9c,11t-CLA↑, 10t,12c-CLA↑, 9c,11t+10t,12c-CLA↑, 18:3 n3↔, 18:3 n6↔, 20:3 n6↔, 20:4 n6↔, 20:5 n3↔, 22:6 n3↔	Bachmair, Wood, Keizer et al. [23]
**Conjugated linoleic acid (Tonalin^®^ WDP 60)** *cis*-9, *trans*-11; *trans*-10, *cis*-12 CLA isomers (50:50 ratios)	Double blind, randomized and placebo controlled study	Healthy sedentary slightly overweight (*n* = 18), were randomly assigned to CLA (*n* = 9) and (*n* = 9) placebo groups	3 g CLA 3 times dailyfor 30 days	WC↔, HC↔, BFM↔, LMB↔, BMI↔, VO_2_ peak↔, TC↔, TG↓, VLDL↓, LDL↓, HDL↔, ApoA↔, ApoB↔, ApoB/ApoA↔, leptin↓, glucose↔, insulin↓, HOMA-IR↔, BChE↓, lipoprotein lipase↑	Bulut, Bodur, Colak and Turnagol [24]
**Conjugated linoleic acid** CLA mixture containing 38.57% of *cis*-9, *trans*-11 isomers, and 39.76% of *trans*-10, *cis*-12 isomers, in an equal proportion (50:50) (Idealfarma, Brazil)	Placebo-controlled and randomized clinical trial	Women with diagnosed MetS (*n* = 14) assigned to placebo (*n* = 7) or glutathione (*n* = 7) groups	3 g CLA/day added to strawberry jam for 90 days	glucose↔, insulin↓, HOMA-IR↔, TG↔, TC↔, LDL↔, HDL↔, SBP↔, DBP↔, BFM↓, BW↔, BMI↔, WC↓	Carvalho, Uehara and Rosa [25]
**Conjugated linoleic acid** CLA mixture of the bioactive isomers 50% *cis*-9, *trans*-11 and 50% *trans*-10, *cis*-12 (Tonalin)	Randomized, double-blind, placebo-controlled trial	Overweight and grade I obese subjects (*n* = 80) divided to CLA (*n* = 40) and placebo (*n* = 40) groups	1.7 g CLA in 200 mL of sterilized milk twice a day for 12 weeks	BW↓, BMI↓, LBM↔, BFM↓, WHR↓, internal organ fat↔, SFM↓, GOT↔, GPT↔, TC↔, TG↔, HDL↔, LDL↔, glucose↔, SBP↔, DBP↔	Chen, Lin, Huang et al. [26]
**Conjugated linoleic acid** - 50:50 mixture of *trans* 10, *cis* 12 and *cis* 9, *trans* 11 CLA (Clarinol^®^ G-80, Lipid Nutrition) - *cis* 9, *trans* 11 (Lipid Nutrition)	Double-blinded, 3-phase crossover clinical trial, placebo-controlled trial	Healthy, overweight, hypercholesterolemic, male volunteers (*n* = 28)	- 3.5 g/d of a 50:50 mixture of t10, c12 and c9, t11 CLA oil (Clarinol G-80, containing 2.8 g of total CLA) - 3.5 g/d of c9, t11 CLA (c9, t11 CLA oil, containing 2.7 g of total CLA) 3 treatment phases of 8 consecutive weeks each alternated with 4 weeks washout periods	BW↔, BMI↔, BFM↔, LBM↔, TC↔, TG↔, VLDL↔, LDL↔, HDL↔, CRP↔, TNF-α↔, IL-6↔, HOMA-IR↔, adiponectin↔, Ox-LDL↔	Joseph, Jacques Plourde et al. [27]
**Creatine monohydrate**	Double-blind, randomized, parallel-group, placebo-controlled trial	Men and women prediagnosed with T2DM, physically inactive for at least 1 yr (*n* = 28), were randomly assigned to the placebo (*n* = 14) and creatine (*n* = 14) groups	5 g/d single dose during lunch for 12 weeks	• HbA1c↓, glucose↓, AUC glucose↓, insulin↔, C-peptide↔, total GLUT-4↔, membrane GLUT-4↑, membrane/total GLUT-4↑, glucose/insulin↔, HOMA-IR↔, HOMA- β ↔, TC↔, TG↔, VLDL↔, LDL↔, HDL↔, apoA1↔, apoA2↔, apoE↔, apoB↔, L(a)↔ • no significant differences were observed between the groups for any physical capacity variable	Gualano, De Salles Painneli, Roschel et al. [28]
**Glutathione** Setria^®^ capsules (Kyowa Hakko USA)	Randomized, double-blinded, placebo-controlled trial	Healthy non-smokers, not taking antioxidant supplements for at least 1 month (*n* = 61) were randomly assigned to one of three treatment GSH low dose (*n* = 20), GSH high dose (*n* = 20) and placebo (*n* = 21)	250 mg/d orally (2 × 125 mg capsules) or 1000 mg/day orally (2 × 500 mg capsules) for 6 months	whole-blood GSH↑, erythrocyte GSH↑, plasma GSH↑, lymphocytes GSH↑, buccal cells GSH↑, (GSSG+GSSP):GSH ratio↓, NK cells cytotoxicity↑, lymphocyte proliferation↔, respiratory burst↔, neutrophil phagocytosis↔	Richie, Nichenametl, Neidig et al. [29]
**Liposomal Glutathione** Tri-Fortify Orange (phosphatidylcholine liposome GSH) (Researched Nutritionals, USA)	Pilot clinical study	Healthy nonsmokers, 50–80 years of age, had no antioxidant supplementation for ≥ 1 month (*n* = 12). Subjects were randomly assigned to low-dose (*n* = 6) or high-dose (*n* = 6) groups	500 mg, *per os* 1000 mg *per os* for 4 weeks	whole-blood GSH↑, erythrocyte GSH↑, plasma GSH, PBMC GSH↑, (GSSG+GSSP):GSH ratio↓, 8-isoprostane↓, NK cell cytotoxicity↑, lymphocyte proliferation↑	Sinha, Sinha, Calcagnotto et al. [30]
**l-Glutathione** Oxition (NTCPharma, Italy)	Double-blinded, randomized placebo controlled crossover study	Healthy male volunteers with one or more cardiovascular risk factors (*n* = 16) randomized to the AB (*n* = 8) and BA (*n* = 8) groups	Oxition 100 mg twice daily for 4 weeks. Each intervention phase lasted 4 weeks with 4 weeks washout period between the two treatments for a total of 12 weeks	ALT↔, GGT↔, TC↓, TG↔, HDL↓, LDL↓, glucose↑, CysGly plasma↑, CysGly reduced blood↔, GSH plasma↔, GSH reduced plasma↔, GSH total blood↔, GSH reduced blood↔, 3-NT↔, MDA↔, PAS↔, PAD↔, HR↔, RHI↔, FRHI↔, augmentation index↔, augmentation index standardized for heart rate of 75 bpm	Campolo, Bernardi, Cozzi et al. [31]
**l-Glutathione** (KOHJIN Life Sciences, Japan)	Open label, single arm, multicenter, pilot trial	NAFLD patients (*n* = 34)	300 mg/d for 4 months by oral administration	BMI↔, glucose↔, IRI↔, HbA1c↑, HDL↔, LDL↔, TG↓, NEFA↓, AST↔, ALT↓, GGT↔, ferritin↓, platelet count↔, type IV collagen 7 s↔, GSH in protein fraction↓, GSH in deproteinized fraction↔, CAP↔, LSM↔	Honda, Kessoku, Sumida et al. [32]
**l-Glutathione** capsules (KOHJIN Co. Ltd., Japan)	Randomized, double-blind, placebo-controlled clinical trial	Healthy, nonsmoking subjects (*n* = 40) men and women assigned to the placebo (*n* = 20) or glutathione (*n* = 20) groups.	1 g (2 × 500 mg/d) administered 15 min before breakfast and dinner for 4 weeks	F2-isoP↔, 8-OHdG↔, GSH↔, GSSG↔,	Allen and Bradley [33]
**α-Lipoic acid**	Randomised, double-blind, placebo controlled, prospective study	T2DM patients (*n* = 23) with diabetic neuropathy and control group- healthy people (*n* = 21)	600 mg lipoic acid/d, 30 min prior to meals for 6 weeks	glucose↔, HbA1c↔, TC↔, TG↔, HDL↔, LDL↔, CRP↔, insulin↔, adiponectin↔	Atmaca, Akbas et al. [34]
**α-Lipoic acid**	Double-blind, placebo-controlled, randomized, clinical trial	Obese patients with NAFLD (*n* = 50) were randomly allocated to the lipoic acid (*n* = 25) and placebo (*n* = 25) groups.	1200 mg/d (2 × 600 mg capsule, one capsule 20 min before breakfast and one capsule 20 min before dinner) plus 400 mg vitamin E/d for 12 weeks	BW↓, BMI↓, WC↓, HC↓, BFM↓, visceral fat↓, total body water↑, free fat mass↑, bone mass↔, ALT↓, AST↓, glucose↓, insulin↓, QUICKI↓, adiponectin↑, MCP-1↔, IL-6↓, ferritin↓, grade of liver steatosis↓	Hosseinpour-Arjmand, Amirkhizi, and Ebrahimi-Mameghani [35]
**α-Lipoic acid** capsules (Puritan’s Pride, USA)	Randomized double-blind placebo-controlled clinical trial study	Pregnant women newly diagnosed with gestational diabetes mellitus (*n* = 60) were divided into drug (*n* = 30) and placebo (*n* = 30) groups	100 mg capsule/d for 8 weeks with lunch	glucose↓, insulin↔, HOMA-IR↓, QUICKI↑, lipoic acid ↑, adiponectin↑, leptin↔, MDA/TAC↓,	Aslfalah, Jamilian, and Khosrowbeygi [36]
**α-Lipoic acid** (produced by Karen Company and capsulated in the School of Pharmacy, Isfahan University of Medical Sciences, Iran)	Randomized, double blind, placebo-controlled clinical trial	Patients with stroke (*n* = 80) were randomly assigned into lipoic acid (*n* = 40) or placebo (*n* = 40) groups	600 mg lipoic acid/d for 12 weeks	SBP↓, DBP↓, glucose↓, insulin↔	Mohammadi, Khorvash, Feizi Askari [37]
**α-Lipoic acid** (produced by Karen Company and capsulated in the School of Pharmacy, Isfahan University of Medical Sciences, Iran)	Randomized, double blind, placebo-controlled clinical trial	Patients who experienced a stroke (*n* = 80) were randomized to the placebo (*n* = 40) and lipoic acid (*n* = 40) groups.	1 capsule containing 600 mg lipoic acid, 1 h before or 2 h after lunch daily for 12 weeks	TG↓, TC↓, LDL↓, HDL↑	Mohammadi, Khorvash, Feizi, Askari [38]
**α-Lipoic acid** capsules	Randomized, double blind, placebo-controlled clinical trial	Patients with T2DM (*n* = 35) were included in lipoic acid group and healthy participants (*n* = 35) were taken as control group	300 mg/d (2 capsules) for 6 months	BW↔, BMI↔, fasting blood glucose↓, postprandial blood glucose↓, HbA1c↓, LDL↓, HDL↑, VLDL↓, TG↓, TC↓, MDA↓, GSH↑, NO↑	Panda, Panda, and Mishra [39]
**Lipoic acid** capsules (Puritan’s Pride, USA)	Randomized double-blind placebo-controlled clinical trial study	Women with gestational diabetes mellitus (*n* = 60) were divided into drug (*n* = 30) and placebo (*n* = 30) groups randomly	100 mg capsule/d for 8 weeks with lunch	glucose↓, α-lipoic acid ↑, ALT↓, AST↔, ALP↔, GGT↓, urea↑, creatinine↔, UA↔, MDA/TAC↓	Aslfalah, Jamilian, Rafiei and Khosrowbeygi [40]
**Taurine** (independent third-party pharmacy)	Single-center, double-blind, randomized, placebo-controlled trial	Untreated participants (*n* = 120) with prehypertension assigned to placebo (*n* = 60) taurine (*n* = 60) groups and age-matched normotensive control subjects without taurine supplementation (*n* = 58)	1.6 g/d for 12 weeks	clinic SBP↓, clinic DBP↓, 24 h ambulatory SBP↓, 24 h ambulatory DBP↓, FMD↑, NMD↑, plasma: H_2_S↑, taurine↑	Sun, Wang, Li et al. [41]
**Taurine** capsules (Landesapotheke, Austria)	Randomized, controlled, double blind trial	Patients with hepatic venous pressure gradient (HVPG) (*n* = 30) were randomly assigned into taurine (*n* = 15) or placebo (*n* = 15) groups	6 g (6 capsules a 1000 mg) for 4 weeks	HVPG↓, FHVP↔, WHVP↔, creatinine↔, BUN↔, bilirubin↔, albumin↔, AST↔, ALT↔, GGT↔, PPT↔, CRP	Schwarzer, Kivaranovic, Mandorfer et al. [42]
**Taurine** (Taisho Pharmaceutical, Japan)	Multicentre, open-label, phase III trial	10 patients with MELAS (mitochondrial myopathy, encephalopathy, lactic acidosis and stroke-like episodes)	9 g/d (participants 25–39 kg BW) or 12 g/d (participants ≥ 40 kg BW) for 52 weeks	• plasma taurine↑, CSF taurine↑, serum lactate↔, CSF lactate↔, serum pyruvate↔, CSF pyruvate↔ • reduction of the annual relapse rate of stroke-like episodes from 2.22 to 0.72 • five patients showed a significant increase in the taurine modification of mitochondrial tRNA^Leu(UUR)^ from peripheral blood leukocytes	Ohsawa, Hagiwara, Nishimatsu et al. [43]

Abbreviations: 3-NT, 3-nitrotyrosine; 8-OHdG, urinary 8-hydroxydeoxyguanosine; ALP, alkaline phosphatase; ALAT, alanine aminotransferase; ALT, alanine transaminase; ApoA1, apolipoprotein A1; ApoB, apolipoprotein B; ApoE, apolipoprotein E; ASAT, aspartate aminotransferase; AST, aspartate transaminase; AUC, area under curve; BChE, butyrylcholinesterase; BFM, body fat mass; BMI, body mass index; BP, blood pressure; BUN, blood urea nitrogen; BW, body weight; CAP, controlled attenuation parameter; CAT, catalase; CLA, conjugated linoleic acid; CML, carboxymethyl lysine; CRP, C-reactive protein; CSF, cerebrospinal fluid; DBP, diastolic blood pressure; F2-isoP, F2-isoprostanes; FA, fatty acids; FFA, free fatty acid; FFM, free fat mass; FMD Flow-mediated dilation; FRHI, Framingham reactive hyperemia index; GDR, glucose disposal rate; GFR, glomerular filtration rate; GGT, gamma-glutamyl transferase; GLUT, glucose transporter; GOT, glutamate oxaloacetate transaminase; GPT, glutamate pyruvate transaminase; GPx, glutathione peroxidase; GSH, glutathione; GSSG, GSH disulfide (GSH oxidation product); GSSP, GSH protein mixed disulfides (GSH oxidation product); HbA1c, glycosylated hemoglobin; HC, hip circumference; HDL, high density lipoprotein cholesterol; HOMA- β, homeostasic model assessment of β-cell function; HOMA-IR, homeostasis model assessment of insulin resistance; HR, heart rate; IL, interleukin; IRAPe, extracellular part of insulin regulated amoinopeptidase; IRI, immunoreactive insulin; LBM, lean body mass; LDL, low density lipoprotein cholesterol; Lp(a), lipoprotein (a); LSM, liver stiffness measurement; MCP-1, monocyte chemoattractant protein; MDA, malondialdehyde; MetS, metabolic syndrome; NEFA, non-esterified fatty acid; NK, natural killer; NMD, nitroglycerin mediated dilation; NO, nitric oxide; Ox-LDL, oxidized LDL; PAD, diastolic blood pressure DBP; PAS, systolic blood pressure SBP; PBMC, peripheral blood mononuclear cells; PC, protein carbonyl; QUICKI, the quantitative insulin check index; RAGE, soluble receptors for advanced glycation end products; RHI, reactive hyperemia index; SBP, systolic blood pressure; SFM, subcutaneous fat mass; SOD, superoxide dismutase; T2DM, type 2 diabetes mellitus; TAC, total antioxidant capacity; TC, total cholesterol; TG, triglycerides; TNF-α, tumor necrosis factor alpha; UA, uric acid; UACR, albumin to creatinine ratio; VLDL, very low density lipoprotein; WC, waist circumference; WHR, waist hip ratio; ↑—value increase; ↓—value decrease; ↔—equivalent values.

**Table 2:** Effects of meat components intake–in vivo studies performed on animals.

Component	Experimental Model	Treatment	Effects	Reference
**Acetyl-l-Carnitine** (Sigma-Tau, Italy)	Pathogen-free male Wistar rats with oxidative stress induced by NaAsO_2_ intoxication (20 mg/kg)	Orally administered 300 mg Acetyl-l-Carnitine/kg, 1 h prior to NaAsO_2_ for 28 days.	• AST↓, ALT↓, LDH↓, bilirubin↓; • oxidant/antioxidant organs status (kidney, liver, heart, lung, brain): GST↑, SOD↑, CAT↑, TBARS↓, -SH↓; • significantly suppressed oxidative organs damage;	Sepand, Razavi-Azarkhiavi, Omidi at al. [44]
**l-carnitine** (Solgar Vitamin and Herb, USA)	Male Wistar Albino rats fed cholesterol rich diet (7.5% cholesterol)	L-carnitine aqueous solution 75 mg/L for 40 days.	TBARS↓, GSH↑, SOD↑, GPx↔, CAT↔	Keskin, Uluisik and Altin [45]
**l-carnitine** (MEPACO, Egypt)	New Zealand rabbits	Diets contained 25, 50 and 100 mg l-carnitine/kg for 4 weeks.	• blood constituents: TC↓, TG↓, HDL↑, LDL↓, VLDL↓, glucose↑; • metabolites: creatinine↑ • plasma enzymes activity: AST↓, ALT↓, ALP↓; • electrolytes: Na↓, K↑, Cl↑; • hormones: T3↑, T4↑, cortisol↓.	Elgazzar, Ghanema and Kalaba [46]
**l-carnitine**	Rats with oxidative stress induced by aspartame intoxication (75 mg/kg or 150 mg/kg)	Oral dose 10 mg l-carnitine/kg for 30 days	• TG↓, TC↓, HDL↑, LDL↓, VLDL↓, ALT↓, AST↓, ALP↓, LDH↓, GGT↓, total proteins↑, albumin↑, CRP↓, TNF-α↓, IL-6↓; hepatic: MDA↓, SOD↑, CAT↑, GPx↑, GSH↑; serum hepatic: MPO↓, XO↓ • more percentage of intact liver cells with undamaged DNA and fewer comet cells • decrased area of damaged cells in liver, obvious improvement liver histology	Hamza, Al-Eisa, Mehana, El-Shenawy et al. [47]
**l-carnitine** (Northeast Pharmaceutical Factory, China)	Male Kunming SPF mice with induced diabetes by high-calorie diet (20% sugar, 18% lard) and two low doses of STZ (100 mg/kg, i.p.) at age of 6 and 9 weeks.	High 250 mg l-carnitine/kg i.g. dose or low 125 mg l-carnitine/kg i.g. dose for 3 weeks.	• BW↓, liver weight↓; • liver: FFA↔, TG↓, L-carnitine↔, Acetyl-L-carnitine↓; • plasma: TG↔; • reduced numer lipid droplet deposits in hepatocytes • recovered mitochondrial damage	Xia, Li, Zhong et al. [48]
**l-Carnosine** (Sigma-Aldrich, USA)	Male Wistar rats with mimic natural agening induced by applying d-galactose subcutaneously as 300 mg/kg, 5 days/week for 2 months	250 mg/kg, i.p. 5 days/week for 2 months	total testosterone↔; testicular: ROS↓, TBARS↓, DC↓, PC↓, AOPP↓, AGE↓, FRAP↔, GSH↔, SOD↔, GPx↔, GST↔	Aydın, Küçükgergin, Çoban et al. [49]
**l-Carnosine** (Sigma-Aldrich, USA)	Male Wistar rats with induced diabetes by high fat diet (60% of total calories from fat) and single STZ injection at a dose of 40 mg/kg BW	250 mg/kg BW i.p. 5 times a Week for last 4 weeks of study	• BW↔, liver weight↔ • serum: glucose↔, HbA1c↔, TG↓, TC↓, ALT↓, AST↓, LDH↓ • serum/plasma: ROS↓, MDA↔, i-MDA↓, AOPP↓, AGE↓, FRAP↔ • hepatic: TG↓, TC↔, ROS↓, MDA↓, PC↓, AOPP↔, AGE↓, FRAP↔, GSH↔, SOD↔, CAT↔, GPx↔; mRNA expression of hepatic SOD↔, GPx↔ • liver histopathologic scoring steatosis↓, lobular inflammation↔ and hepatocyte ballooning↓	Aydın, Bingül, Küçükgergin et al. [50]
**l-Carnosine** (Sigma-Aldrich, USA)	Male Wistar rats with induced diabetes by high fat diet (34.3–60% fat of total calories) and STZ injection at a dose of 40 mg/kg BW	250 mg/kg BW i.p. 5 times a week for 4 weeks	• BW↔, kidney weight↔ • blood: glucose↔, HbA1c↔, TG↓, TC↓, • serum: BUN↓, creatinine↓, total protein↔, albumin↔ • kidney: ROS↓, MDA↓, PC↓, AOPP↓, AGE↓, FRAP↔, GSH↔, SOD↔, CAT↔, GPx↔; mRNA expression of kidney SOD↔, GPx↔ • Histopathologic examination of kidney tissue showed normal appearance of glomeruli and tubules in all rat groups	Aydın, Küçükgergin, Bingül et al. [51]
**l-Carnosine** (Sigma-Aldrich, USA)	Aged (20 months-of-age) male Wistar rats	250 mg/kg/5 days per week; i.p. for 2 months	• serum/plasma: AGE↓, PC↓, AOPP↓, MDA↓, FRAP↔, ROS↓ • liver: AGE↓, PC↓, AOPP↓, MDA↓, FRAP↔, ROS↓	Bingül, Yılmaz, Aydın et al. [52]
**Coenzyme-Q_10_** (Mepaco company, Egypt).	Male albino rats fed cholesterol rich diet (5% cholesterol)	1mg coenzyme Q10/rat by oral gavage for 4 months	• TG↓, TC↓, HDL↑, LDL↓, SOD↑, CAT↑, GPx↑, MDA↓, • amelioration histological and biochemical structure of cerebellal cortex	El-Haleem, Yassen, and Raafat [53]
**Creatine monohydrate**	Male Sprague-Dawley rats with NAFLD induced by high-fat liquid diet with 71% of energy derived from fat	Free access to food diet with 1% (*w*/*v*) creatine monohydrate throughout the 3 weeks	• BW↔, • liver: fat↓, TG↓, TC↓, TBARS↓, SAM↑, SAH↔, SAM/SAH↑, phosphatidylcholine↔, phosphatidylethanolamine↑; mRNA levels: Pemt↔, PPARα↑, CD36↓, CPT1a↑, LCAD↑, Bhmt↓, Gnmt↓, MGAT↓ • plasma: glucose↔, insulin↔, creatine↑, GAA↓, Hcy↔, Cys↑, • kidney: AGAT↓	Deminice, da Silva, Lamarre et al. [54]
**Creatine monohydrate**	Male Wistar rats with nonalcoholic steatohepatitis (NASH) induced by choline-deficient diet	2% (*w*/*v*) creatine monohydrate in diet (free access to food) for 4 weeks	• BW↔, food intake↔ • plasma: creatine↑, Hcy↓, methionine↔, Cys↔, phosphatidylcholine↔, ALT↓, TNF-α↓ • liver: fat↓, TG↓, TC↓, creatinie↑, SAM↔, SAH↑, SAM/SAH↔, phosphatidylcholine↔, MDA↓, GSH↑, GSH/GSSG↑, TNF-α↓, PPARγ↔, • mRNA genes expression: - methionine metabolism: Bhmt1↑, Cbs↔, Pemt↔, Gnmt↑ - phospholipids metabolism: Chka↔, Chkb↔, ChDh↓, Pcyt1a↔ - MTP↔ - transcription factors: PPARα↓, PPARγ↔, - fatty acid oxidation genes: UCP2↓, PGC1a↔, LCAD↑, CPT1a↓, FABP3↔, HAD↔ • kidney: AGAT↓	Deminice, de Castro, Francisco et al. [55]
**Creatine monohydrate**	Sprague–Dawley rats with NAFLD induced by HFD (0.82 kcal/g protein, 3.24 kcal/g fat and 1.43 kcal/g carbohydrate for a total of 5.49 kcal/g)	2% creatine monohydrate in diet (20 g/kg) for 4 weeks	• BW↔, calorie intake↔, • liver: weight↔, TG↓, cholesterol ester↓, MTTP↔, • liver cytokines: Eotaxin↔, EGF↔, Fractalkine↔, IFN-γ↔, IL-1α↔, IL-1β↔, IL-2↔, IL-4↔, IL-5↔, IL-6↔, IL-10↓, IL-12(p70) ↔, IL-13↔, IL-17A↔, IL-18↔, IP-10↔, GRO/KC↔, TNF-α↔, G-CSF↔, GM-CSF↔, MCP-1↔, leptin↔, LIX↔, MIP-1α↔, MIP-2↔, RANTES↔, VEGF↔. • plasma: appearance over time TG↔, ApoB48↑, ApoB100↔; fasting: TG↔, ApoB48↔, ApoB100↔; AUC: TG↔, ApoB48↔, ApoB100↔. • mitochondrial respiratory chain complexes: VDAC loading control↑, VDAC loading control: complex I↔, II↔, III↔, IV↔, V↔; PDI loading control: complex I↔, II↑, III↔, IV↔, V↔; ND6 DNA↔, ATP6 DNA↔	da Silva, Leonard and Jacobs [56]
**α-Lipoic acid** (Hi-Media chemicals, India)	Male Sprague-Dawley albino rats with fructose-induced experimental cataract (10% *w*/*v* fructose solution in drinking water-equivalent to a diet containing 48–57% fructose) for 8 weeks	20 or 40 mg lipoic acid/kg/d orally by gavage for 8 weeks	• MAP↓, glucose↓, lens: GPx↑, CAT↑, SOD↑, GSH↑, MDA↓, total proteins↑, Ca^2+^ ATPase activity↑, Ca^2+^↓, • potentially reduced progression of cataract formation: stage of cataract↓, delayed progression of cataract formation	Khan, Choudhary, Vishwakarma et al. [57]
**α-Lipoic acid** powder (Sigma, USA)	Wistar rats with alloxan induced diabete	100 mg lipoic acid/kg/d BW i.p. injection for 6 weeks	• serum: GPx↑, CAT↑, MPO↓, MDA↓, GSH↑, glucose↓, urea↓, creatinine↓ • liver: : GPx↔, CAT↑, MPO↓, MDA↓, GSH↑, • kidney: : GPx↑, CAT↑, MPO↓, MDA↓, GSH↑, mRNA levels: SOD↑, CAT↑ GPx↑, • histopathological lesions such as increased glomerularvolume and lymphocyte infiltration were attenuated	Jamor, Ahmadvand, Ashoory and Babaeenezhad [58]
**α-Lipoic acid**	C57BL6 mice with obesity induced by high-fat diet (60% kcal% fat)	0.2% lipoic acid in diet for 12 weeks	BW↓, food intake↓, caloric intake↓, % body fat↔, LBM↓, BFM↓	Panzhinskiy, Bashir, Bagchi and Nair [59]
**α-Lipoic acid** powder (Sigma, USA)	Male Sprague Dawley rats with diabetes inducted with injection of 100 mg/kg alloxan	100 mg lipoic acid/kg was injected i.p. daily for 6 weeks	glucose↓, TG↓, TC↓, HDL↑, LDL↓, VLDL↓, PON1↑	Jamor, Ahmadvand, Birjandi and Sharafabad [60]
**α-Lipoic acid**	Diabetic Goto-Kakizaki rats fed HFD (7.5% cocoa butter, and 1.25% cholesterol)	50 mg/kg BW i.p., 3 days/week for 3 months	• BW↔, liver weight↓, fasting blood glucose↓, blood glucose 2 h after load↔, TC↓, non-HDL↓, TG↓, albumin↔, T-Bilirubin↔, AST↓, ALT↔, ALP↓, GGT↓, HEF↑, MDA↓, 8-OHdG↓, UA↓ • liver: TC↓, TG↓, GPx↑, GRd↑, MDA↓, GSH↑, Nrf2↑, TNF-α↓,	Sena, Cipriano, Botelho and Seiça [61]
**Liposomal Glutathione** (8.25% GSH (84.5 mg/mL), 75.15% deionized water, 15% glycerin, 1.5% lecithin, and 0.1% potassium sorbate (% *w/w*)	Atherosclerotic apolipoprotein E-deficient (E^0^) mice	12.5 or 50 mg/kg/d in drinking water for 2 months	• TC↓, HDL↓, TG↑, glucose↔, AAPH induced serum lipid peroxidation↓, • mouse peritoneal macrophages (MPM): GSH↑, PON2 lactonase activity↑, total peroxides↓, LDL uptake↓, Ox-LDL uptake↓, cholesterol biosynthesis↓, HDL-mediated macrophage cholesterol efflux↑, TC↓, atherosclerotic lesion area↓	Rosenblat, Volkova, Coleman and Aviram [62]
**Liposomal Glutathione** “ReadiSorb” glutathione (Your Energy Systems, LLC, USA)	Male, New Zealand white rabbits	Orally administered 5 mL of liposomal glutathione (containing approximately 428.8 mg of GSH) for 3, 7 or 14 days	LVEDP↓; LVDP↓; CPP↓; total GSH: heart↑, liver↑, brain↔; cTnI↔; heart MDA↔	Lauver, Kaissarian and Lucchesi [63]
**Peptides (protein hydrolysate, Phe-Gln-Pro and Phe-Gln-Pro-Ser)** protein hydrolysate from meat of *Kacang* goat (*Capra aegagrus hircus*) was obtained by Protamex^®^ and Flavourzyme^®^ digestion	Male SHR	Single oral administration: - 0.01 or 0.1 g hydrolysate *Kacang* goat meat/kg BW - 0.00195 g Phe-Gln-Pro/kg BW - 0.00239 g Phe-Gln-Pro-Ser/kg BW	• after administering 0.01 g or 0.1 g hydrolysate/kg BW highest reduction of SBP was 19.3 or 26.9 mmHg, occurred at 6 h after administration. SBP was still significantly lower than that of the control group after 24 h. • Phe-Gln-Pro showed the highest reduction of SBP by 12.6 mm Hg at 6 h • Phe-Gln-Pro-Ser showed the highest reduction of SBP by 10.6 mmm Hg at 8 h after administration • SBP 24 h after pure peptides administration was not different to the controls	Mirdhayati, Hermanianto, Wijaya et al. [64]
**Peptides** Three sample extracts of pooled fractions from Spanish dry-cured hams	Male SHRs	Single oral administration 4.56 mg of sample 1/kg BW or 1.48 mg of sample 2/kg BW or 8.7 mg of sample 3/kg BW by gastric intubation with a metal tube	• All samples decrase SBP: - sample 1 by 33.1 mm Hg and 38.38 mm Hg after 4 and 6 h; - sample 2 by 27.48 mm Hg after 6 h - sample 3 by 23.56 mm Hg at 6 h after oral administration. • In all cases SBP returned to pretreatment values after 24 h.	Escudero, Aristoy, Nishimura et al. [65]
**Peptides (RPR, KAPVA and PTPVP)** peptides identified in pork meat hydrolysate after in vitro digestion	Male SHRs	Single administration of distilled water peptide suspension 1 mg peptide/kg of BW by gastric intubation.	• analysed peptides decrase mean SBP compared with the control SHRs: - RPR decrease 33.21, 28.81 and 21.16 mm Hg at 6, 8 and 4 h after administration - KAPVA decrease 19.1 and 33.72 at 4 and 6 h after administration - PTPVP decreased by 24.52 and 25.66 mm Hg at 4 and 6 h after administration • in all cases SBP returned to pretreatment values after 24 h.	Escudero, Toldrá, Sentandreu et al. [66]
**Peptides (KRVITY, Lys-Arg-Val-Ile-Gln-Tyr; VKAGF, Val-Lys-Ala-Gly-Phe)** identified in pork loin muscle after extraction and pepsin hydrolysis	SHRs	10 mg KRVITY or VKAGF/kg BW with a metal oral syringe	• KRVITY decrease SBP by 12 mmHg in 3 h and 23 mmHg in 6 h after oral administration • VKAGF decrease SBP by 12 mmHg in 3 h and 17 mmHg in 6 h after oral administration	Muguruma, Ahhmed, Katayama et al. [67]
**Peptides (YYRA, Tyr-Tyr-Arg-Ala)** identified in chicken bone after extraction and hydrolysis with pepsin	SHRs	Single oral administration 10 mg/kg BW administered orally by intubation.	SBP decrase significantly over a short period of time 3 h from 3rd to 6th h	Nakade, Kamishima, Inoue et al. [68]
**Peptides** low fraction hydrolysate from chicken legs collagen obtained by extraction and digestion with protease	Male SHRs	3 g hydrolysate/kg BW single administration or long-term administration for 4 weeks	• after single administration reduction in blood pressure was observed from 4 to 8 h • long-term administration showed that there was a reduction in from 2nd to 4th week of the study	Saiga, Iwai, Hayakawa et al. [69]
**Taurine** (Sigma Chemical Co., USA)	Male albino rats (*Rattus norvegicus*) i.p. injected with 5-fluorouracil (20 mg/kg BW/day) for 7 days.	50 mg/kg BW/day for 21 days: 7 days alone, 7 days parallel with i.p. injections with 5-fluorouracil, 7 days alone	• BUN↓, creatinine↓, UA↓, SOD↑, CAT↑, GPx↑, MDA↓, GGT↑, ALP↑ • reversed most histological and ultrastructural alterations in kidney tissues	Yousef and Aboelwafa [70]
**Taurine**	Male Wistar rats fed high fructose diet (60% fructose)	2% taurine solution *ad libitum* for 30 days	BW↔, SBP↓, kallikrein: heart↑, kidney↑, plasma↑, urine↑; sodium: plasma↓, urine↑	Nandhini and Anuradha [71]
**Taurine** (Taisho Pharmaceutical, Japan)	Male Golden Syrian hamsters fed high-fat diet (0.05% cholesterol and 10% coconut oil).	Taurine dissolved in drinking water at 1% (*w*/*v*) was freely available for 14 days	• BW↔, TC↓, non-HDL↓, HDL↔, TG↓, phospholipids↓, ACAT↓, HMG–CoA reductase↔, cholesterol 7a–hydroxylase↑, • up-regulation LDL receptor activity • acceleration receptor-mediated LDL turnover	Murakami, Kondo, Toda et al. [72]
**Taurine** (Sigma Chemicals, USA)	Male Wistar rats with oxidative injuries induced by Fipronil supplementation 19.4 mg/kg for 5 days (6–10th day of the experiment).	Oral dose 50 mg/kg daily (5 days before and 5 days along with Fipronil supplementation)	• liver: MDA↓, NO↓, GSH↑, GPx↑, SOD↑, CAT↑, AST↓, ALT↓, ALP↓, LDH↓, TC↓, • kidney: MDA↓, NO↓, GSH↑, GPx↑, SOD↑, CAT↑, urea↓, creatinine↓ • amelioration and normalization of the harmful effects of Fipronil on hepatorenal injury	Abdel-Daim, Dessouki, Abdel-Rahman et al. [73]
**Taurine** (Sigma–Aldrich Chemical Company, USA)	Male Wistar rats with diabetes and testicular damage induced by one i.p. injection of 50 STZ mg/kg BW	100 mg/kg BW daily, via oral gavage, for 6 weeks.	• glucose↓, insulin↑, testis weight/BW↑, MDA↓, protein carbonylation↓, GSH/GSSG↑, SOD↑, CAT↑, TNF-α↓, IL-1β↓, IL-6↓, MCP-1↓, ICAM-1↓, VCAM-1↓, testosterone↑, 3β-HSD↑, 17β-HSD↑, SDH↑ • testicular tissue: **ER stress related pathway**: calpain-1↓, cleaved Caspase-12↓, p-PERK↓, p-eIF2α/total eIF2α↓, CHOP↓, Grp78↓; **NFκB mediated pathway**: nuclear NFκB↓, cytosolic NFκB↑, phospho and total I ĸBα↓; **mitochondria dependent apoptotic pathways**: Bax/Bcl-2↓, cytosolic cytochrome-C↓, mitochondrial cytochrome-C↑, cleaved Caspase-9↓, cleaved Caspase-3↓, cleaved PARP↓ • treatment with taurine improve histological alterations like loss of spermatids, disappearance of testicular cells like Leydig and Sertoli cells, sloughing of centrally located spermatozoa and the disruption of germinal epithelium.	Ghosh, Chowdhury, Das et al. [74]
**Taurine** (Sigma-Aldrich, USA)	Male BALB/c mice with Colistin (15 mg/kg/d, i.p. for 7 consecutive days) associated renal injury	500 or 1000 mg/kg/d, i.p for 7 consecutive days	• BUN↓, creatinine↓, kidney: ROS↓, TBARS↓, TAC↑ GSSG↓, GSH↑, GSH/GSSG↑, histopathological SQS↓ • mitochondrial: dehydrogenases↑, swelling↓, depolarization↓, ATP↑, TBARS↓, GSH↑, GSSG↓, GSH/GSSG↑	Heidari, Behnamrad, Khodami et al. [75]
**Taurine** (Sigma Chemical Co., USA)	Male Wistar rats with hypertension induced by L-NAME at 40 mg/kg BW p.o. daily	100 and 200 mg/kg p.o. for 28 days	• SBP↓, DBP↓, MAP↓, BW↔, OSI of the testes↔, OSI of the epididymis↔, ACP↑, ALP↑, LDH↑, LH↑, FSH↑, testosterone↑, • testes: SOD↑, CAT↑, GPx↑, GSH↑, H_2_O_2_↓, MDA↓, MPO↓, NO↑ • epididymis: SOD↔, CAT↑, GPx↑, GSH↔, H_2_O_2_↓, MDA↓, MPO↓, NO↑ • sperm: testicular sperm number↑, epididymal sperm number↑, motility↑, viability↔, abnormalities↔	Adedara, Alake, Adeyemo et al. [76]
**Taurine** (Sigma-Aldrich, USA)	Male Wistar albino rats with malathion induced toxicity (27 mg/kg orally)	0.5 mL taurine solution at dose of 50, 100, and 200 mg/kg orally for 30 days	• blood: MDA↓, GSH↑ • erythrocyte: SOD↓, CAT↔ • serum: AChE↑ • liver: MDA↓, GSH, SOD↓, CAT↓, AChE↑, mRNA levels: IFN-γ↓, NFĸB↓, TNF-α↓, IL-1β↓, • testis: MDA↓, GSH↑, SOD↓, CAT↓, • brain: MDA↓, GSH↑, SOD↓, CAT↓, • kidney: MDA↓, GSH↑, SOD↓, CAT↓, • preventive action against malathion-induced histopathological changes in rat tissues.	Ince, Arslan-Acaroz, Demirel et al. [77]
**Taurine** (Sigma-Aldrich, USA)	Male Wistar rats with diabetes induced by a single i.p. injection of 40 mg STZ/kg BW	50 mg/kg BW for 60 days	AChE↓, GnRH↓, TRH↑, T3↑, T4↑, TSH↓, testosterone↑, FSH↓, LH↓, sperm count↑, abnormal sperms↓, motility↑, • brain: MDA↓, SOD↑, CAT↑, • thyroid: MDA↓, SOD↑, CAT↑, • testis: MDA↓, SOD↑, CAT↑ • marked repairing of testicular abnormalities and a maximum healing effect against STZ induced testicular damage	Mohamed and Gawad [78]
**Taurine** (Sigma-Aldrich, USA)	Male Wistar rats with cognitive impairment induced by intracerebroventricular STZ injection at a dose of 3 mg/kg	40, 60 and 120 mg/kg p.o. by gavage for 28 days	• BW↔, • cortex: GSH↑, MDA↓, NO↓, SOD↑, AChE↓, BChE↓, TNF-α↓, IL-1β↓, ROCK-II↓, GSK-3β↔, ChAT↔ • hippocampus: GSH↑, MDA↓, NO↓, SOD↑, AChE↓, BChE↓, TNF-α↓, IL-1β↓, ROCK-II↓, GSK-3β↔, ChAT↑ • improved behavioural parameters: escape latency↓, time spent in target quadrant↑, retention transfer latency in elevated plus maze test↓, transfer latency in passive avoidance test↑	Reeta, Singh and Gupta [79]

Abbreviations: 17β-HSD, 17β-hydroxysteroid dehydrogenase; 3β-HSD, 3β-hydroxysteroid dehydrogenase; 8-OHdG, urinary 8-hydroxydeoxyguanosine; AAPH, 2,2-azobis 2, amidinopropane hydrochloride; ACAT, acyl-CoA cholesterol acyltransferase; AChE: acetylcholinesterase; ACP, acid phosphatase; AGAT, arginine:glycine amidinotransferase; AGE, advanced glycation end products; ALP, alkaline phosphatase; ALT, alanine aminotransferase; ALT, alanine transaminase; AOPP, advanced oxidised protein products; ApoA1, apolipoprotein A1; ApoB, apolipoprotein B; AST, aspartate aminotransferase; AST, aspartate transaminase; ATP6, mitochondrially encoded ATP synthase membrane subunit 6; AUC, area under curve; Bax, pro apoptotic protein; BChE, butyrylcholinesterase; Bcl-2, B-cell lymphoma 2; BFM, body fat mass; Bhmt, betaine-homocysteine S-methyltransferase; BUN, blood urea nitrogen; BW, body weight; CAT, catalase; Cbs, cystathionine beta synthase; CD36, scavenger receptor that functions in high affinity tissue uptake of long chain fatty acids; ChAT, choline acetyltransferase; ChDh, choline dehydrogenase; Chka, choline kinase alpha; Chkb, choline kinase beta; CHOP, C/EBP homologous protein; CPP, coronary perfusion pressure; CPT1a, carnitine palmitoyltransferase 1a; CRP, C-reactive protein; cTnI Cardiac-specific troponin I; Cys, cysteine; DBP, diastolic blood pressure; DC, diene conjugate; EGF, epidermal growth factor; eIF2α, eukaryotic initiation factor 2α; FABP3, fatty acid binding protein 3; FFA, free fatty acid; FRAP, ferric reducing anti-oxidant power; FSH, reproductive hormone; GAA, guanidinoacetic acid; G-CSF, granulocyte colony stimulating factor; GGT, gamma-glutamyl transferase; GM-CSF, granulocyte macrophage colony stimulating factor; Gnmt, glycine N-methyltransferase; GnRH, gonadotropin releasing hormone; GPx, glutathione peroxidase; GRd, glutathione reductase; GRP78, 78 kDa glucose regulated protein; GSH, glutathione; GSK-3β, glycogen synthase kinase-3β; GSSG, GSH disulfide (GSH oxidation product); GST, glutathione transferase; HAD, hydroxyacyl CoA dehydrogenase; HbA1c, glycosylated hemoglobin; Hcy, homocysteine; HDL, high density lipoprotein cholesterol; HEF, hepatic extraction fraction; HFD, high fat diet; HMG-CoA, 3–hydroxy–3–methylglutaryl coenzyme A; i.g., intragastric; i.p., intraperitoneal; i.v., intravenous; ICAM-1, intercellular adhesion molecule; IFN-γ, interferon gamma; IL, interleukin; i-MDA endogenous and AAPH-induced malondialdehyde; IP-10, IFN-γ induced protein 10; LBM, lean body mass; LCAD, long-chain acyl-CoA dehydrogenase; LDH, lactate dehydrogenase; LDL, low density lipoprotein cholesterol; LH, reproductive hormone; LIX, lipopolysaccharide-induced CXC chemokine; L-NAME: N-nitro L-argininemethyl-ester; LVDP, left ventricular developed pressure; LVEDP, left ventricular end diastolic pressure; MAP, mean arterial pressure; MCP-1, monocyte chemoattractant protein; MDA, malondialdehyde; MGAT, mannosyl (alpha-1,3-)-glycoprotein beta-1,2-N-acetylglucosaminyltransferase; MIP, macrophage inflammatory proteins; MPO, myeloperoxidase; MTP, microsomal triglyceride transfer protein; MTTP, microsomal triglyceride transfer protein; ND6, NADH dehydrogenase, subunit 6 (complex I); NFκB, nuclear factor kappa; NO, nitric oxide; Nrf2- nuclear factor E2 (erythroid-derived 2)-related factor-2; OSI, organo somatic indices; Ox-LDL, oxidized LDL; PARP, poly (ADP-ribose) polymerase; PC, protein carbonyl; Pcyt1a, phosphate cytidylyltransferase 1; PDI, protein disulfide isomerase; Pemt, phosphatidylethanolamine N-methyltransferase; PERK, Protein kinase R like endoplasmic reticulum kinase; PGC1a, peroxisome proliferator-activated receptor gamma, coactivator 1 alpha; PON1, paraoxonase 1; PON2, paraoxonase 2; PPAR, peroxisome proliferator activated receptor; RANTES, regulated on activation, normal T-cell expressed and secreted; ROCK-II, rho kinase II, ROS, reactive oxygen species; SAH, S-adenosylhomocystein; SAM, S-adenosylmethionine; SBP, systolic blood pressure; SDH, sorbitol dehydrogenase; SH, sulfhydryl group; SHR, spontaneously hypertensive rats; SOD, superoxide dismutase; SPF, specific pathogen free; SQS, semi-quantitative score; STZ, streptozotocin; T3, triiodothyronine; T4, thyroxine; TAC, total antioxidant capacity; TBARS, thiobarbituric acid reactive substances; TC, total cholesterol; TG, triglycerides; TNF-α, tumor necrosis factor alpha; TRH, thyroid releasing hormone; TSH, thyroid stimulating hormone; UA, uric acid; UCP2, uncoupling protein 2; VCAM-1, vascular cell adhesion molecule; VDAC, voltage dependent anion channel; VEGF, vascular endothelial growth factor; VLDL, very low density lipoprotein; XO, xanthine oxidase; ↑—value increase; ↓—value decrease; ↔—equivalent values.

**Table 3:** Contents of l-carnitine and taurine in meat and animal origin products.

Product	Content (mg/100 g)	
	l-carnitine Kalpana [82]; Dayanand et al. [85]	Taurine Lourenco and Camilo [86]
Kangaroo meat	637	n.e.
Horse meat	423	n.e.
Lamb	190	43.8
Veal	n.e.	39.8
Steak tartare	183	n.e.
Beef	139–143	43.1
Pork	25–60.8	61.2
Beef ribs	226	n.e.
Pork ribs	40.2	n.e.
Duck, fillet	73.2	n.e.
Turkey fillet	51.4–200	29.5
Chicken fillet	13–34.4	17.8
Pheasant breast	13.5	n.e.
Beef liver	15.6	69
Pork liver	10.7	89
Poultry liver	n.e.	110
Eggs	0.8	n.e.
Cow milk, 1.5% fat	40	1
Goat milk	n.e.	7
Buttermilk	38.5	n.e.
Sour cream	19.7	n.e.
Hard cheese	2.8	n.e.
Brie	5.85	n.e.
Feta	14.9	n.e.
Tuna	n.e.	68
Cod	n.e.	31
Atlantic salmon	n.e.	130
Oysters	n.e.	396

n.e.—not evaluated.

**Table 4:** Carnosine contents in animal origin foodstuffs.

Products	Content (mg/100 g) Aristoy and Toldra [92]
Pork loin	313
Pork ham	449
Beef loin	375
Lamb shoulder	39.3
Chicken breast	180
Chicken thigh	63
Turkey wings	66
Salmon	0.53
Trout	1.6
Sardine	0.1

**Table 5:** Contents of choline, alpha-lipoic acid and conjugated linoleic acid (CLA) in meat and animal origin products.

Product	Choline (mg/100 g) Patterson et al. [98]	Alpha-Lipoic Acid (mg/100 g) Schmid [4]	CLA (mg/g fat) Koba and Yanagita [117]
Poultry, chicken liver	190	n.e.	n.e.
Poultry, turkey liver	220	n.e.	n.e.
Poultry, turkey heart	130	n.e.	n.e.
Poultry, turkey meat	n.e.	n.e.	2.0–2.5
Poultry, broiler meat	66	n.e.	0.7–1.5
Turkey sausage, fresh	51	n.e.	n.e.
Pork sausage, fresh	53	n.e.	n.e.
Poultry frankfurters	51	n.e.	n.e.
Ground pork	69	0.02–0.03	0.6
Pork, neck	79	0.02–0.04	n.e.
Pork, liver	n.e.	0.06–0.08	n.e.
Pork, heart	n.e.	0.11–0.16	n.e.
Lean beef, ground	66	n.e.	4.3
Beef, liver	330	0.06–0.11	n.e.
Beef, neck	100	n.e.	n.e.
Beef, heart	n.e.	0.07–0.10	n.e.
Mutton	n.e.	n.e.	5.6
Veal, muscle	n.e.	0.01–0.02	2.7
Veal, liver	310	0.03–0.05	n.e.
Veal, heart	n.e.	0.05–0.07	n.e.
Lamb, liver	n.e.	0.07–0.08	n.e.
Lamb, muscle	n.e.	0.02–0.04	4.3–19.0
Lamb, heart	n.e.	0.05–0.07	n.e.
Reindeer meat, ground	150	n.e.	n.e.
Whole eggs, fresh	250	n.e.	n.e.
Egg yolk	680	n.e.	0.6
Cheddar	17	n.e.	4.0–5.3
Whole milk, 3.25% fat	14	n.e.	5.5
Feta	n.e.	n.e.	4.9
Butter	19	n.e.	4.7
Cream	n.e.	n.e.	4.6–7.5

n.e.—not evaluated.

**Table 6:** Content of glutathione (GSH) in food.

Product	Content (mg/100 g) Bukowska [130]
Cooked ham	23.3
Fried chicken	13.1
Fried beef	17.5
Fried bacon	5
Beefsteak	12.3
Tuna in oil	1.1
Spinach	11.4
Carrot	5.9
Potatoes	11
Asparagus	28.3
Oranges	7.3
Bananas	3.3

**Table 7:** Contents of coenzyme Q10 and creatine in meat and animal origin products.

Product	Content (mg/100 g)	
	Coenzyme Q10 Borekova et al. [159]	Creatine Schmid [4]
Beef	3.65	n.e.
Beef, semitendinosus muscle	n.e.	401
Lamb	n.e.	278–511
Pork, ham	2	247–374
Reindeer meat	15.7	n.e.
Chicken	1.4	n.e.
Beef, heart	11.3	298
Beef, liver	3.9	16
Pork, heart	12.6	n.e.
Pork, liver	2.27	n.e.
Herring	n.e.	650–1000
Salmon	n.e.	450
Tuna	n.e.	400
Cod	n.e.	300
Eggs	0.12	n.e.
Milk, 1.5% fat	0.01	n.e.
Edam cheese	0.12	n.e.

n.e.—not evaluated.
